# Sex influences on tumor innervation

**DOI:** 10.1186/s13293-026-00887-9

**Published:** 2026-04-10

**Authors:** Sarah M. Barclay, Jeffrey Barr, Oduduabasi Isaiah, Mangalam Bajpai, Hailey Bullard, Ethan Neufeld, Craig C. Welbon, Payal Ghosh, Destiny S. Brockhaus, William C. Spanos, Paola D. Vermeer

**Affiliations:** 1 Sanford Research Institute, 2301 East 60th Street north, Sioux Falls, SD USA; 2 Biomedical and Translational Sciences, University of South Dakota, 414 E. Clark Street, Vermillion, SD USA; 3 Biomedical and Veterinary Sciences department, Virginia Tech, 205 Duck Pond Drive, Blacksburg, VA USA; 4 Eastern Mennonite University, 1200 Park Road, Harrisonburg, VA USA; 5 Sanford School of Medicine, Department of Surgery, University of South Dakota, 414 E. Clark Street, Vermillion, SD USA

## Abstract

Peripheral solid malignancies are infiltrated by nerves. Using syngeneic cell lines, we set out to define whether sex influences tumor innervation in oral cancers and melanoma. Tumors grew significantly slower in females and were significantly more innervated than in males. In oral cancer, tumor-infiltrating nerves connect to a pre-existing neural circuit that enters the brain and impacts behavior. Thus, we evaluated the impact of sex on this circuit and assessed the behavioral consequences. We found no differences in the timing or extent of behavioral declines between oral tumor-bearing males and females. Moreover, in untreated mice, metastasis did not differ by sex. Treatment of oral tumors with cisplatin and radiation slowed tumor growth in male and female mice but significantly increased tumor innervation with females harboring the greatest innervation density. Treated females also harbored significantly increased metastases in their lungs compared to treated males. Treatment improved tumor-associated behavioral changes. While the tumor-brain circuit did not differ between males and females, the activation status of neurons and glia in projection areas significantly increased on the ipsilateral side. This heightened activation was attenuated following cisplatin/radiation treatment. These studies indicate that peripheral malignancies activate neurons and glia in central nervous system (CNS) projection areas, and that standard-of-care therapy attenuates this, which correlates with improved behavior. These findings emphasize that sex influences peripheral tumor-infiltrating nerves and brain function (via the tumor-brain circuit) and suggest that severing or silencing this circuit could improve patient outcomes.

**Supplementary Information:**

The online version contains supplementary material available at 10.1186/s13293-026-00887-9.

## Abstract (plain language summary)

Tumors consist of many cell types: malignant cells, immune cells, fibroblasts and vascular cells/structures; the presence of nerves within tumors is now widely accepted. Using a mouse model of head and neck cancer, we previously showed that tumor-associated nerves are integrated into a circuit that extends into the brain. Neurons within this tumor-to-brain circuit undergo changes that result in altered behaviors. However, these studies used only male mice. Therefore, to determine if sex differences exist in this circuit, we now extend our findings to include female mice. Because head and neck cancer patients typically receive chemotherapy and radiation, we also analyzed the impact of this treatment on the tumor-to-brain circuit, tumor growth, metastasis and behavior. We show that head and neck tumors grow more slowly but are more densely innervated in females than males. The tumor-to-brain circuit and behavioral changes associated with cancer, however, remain similar between the sexes. We found that treatment increases innervation of tumors in both sexes, with treated females harboring the most innervation. Treatment increased lung metastasis in females compared to males, though behavioral responses were similar between the sexes. These findings emphasize that sex influences the density of nerves within head and neck tumors, that treatment amplifies this difference. This correlates with increased metastasis in females. Our findings suggest that females with head and neck cancer may be exquisitely responsive to nerve-focused therapies in oncology. While not currently available, future inclusion of these therapies may have a significant patient impact.

**Supplementary Information:**

The online version contains supplementary material available at 10.1186/s13293-026-00887-9.

## Highlights

- Using syngeneic mouse models of human papillomavirus negative and positive head and neck cancer as well as melanoma, we show that tumors in female mice grow significantly slower but are significantly more innervated than in the male counterparts.
- This increased nerve density at the primary tumor site did not alter the tumor-to-brain circuit and, consistent with this, no behavioral differences between tumor-bearing male and female mice were evident.
- Chemoradiotherapy potentiated tumor innervation in both sexes with female treated tumors harboring the greatest extent of innervation; moreover, following treatment, female mice had significantly more metastatic lesions in their lungs compared to males.
- Despite treatment-induced increased tumor innervation, calcium imaging studies of tumor-infiltrating neurons indicated that treatment decreased neuronal activity as compared to neurons from untreated tumor-bearing animals.
- These findings identify sex differences in head and neck tumor innervation and treatment response and suggest that females may be exquisitely responsive to inclusion of nerve-targeted therapy.

**Supplementary Information:**

The online version contains supplementary material available at 10.1186/s13293-026-00887-9.

## Introduction

The presence of nerves within cancers has been recognized since the late 1800’s [1, 2]. However, over a century passed before convincing studies in esophageal, fibrosarcoma, and pancreatic cancers [3–5] reignited interest in this phenomenon. Over the past decade, the influence of tumor-infiltrating nerves on cancer initiation and progression has become a major research focus. Immunohistochemical (IHC) staining of many malignancies (breast, prostate, pancreatic, lung, liver, ovarian, colon, head and neck, cervical, and melanoma) for neuronal markers has unequivocally revealed the presence of axons and axon bundles within tumors indicating that most, if not all, peripheral malignancies are innervated [6–15]. Electrophysiologic studies show that tumor-infiltrating nerves remain functional at the tumor bed, while molecular studies indicate the nerve-released factors, like neurotransmitters and neuropeptides, impact disease progression via direct and indirect mechanisms [15–17]. Evaluation of sex in the context of many malignancies indicates the presence of differences among patients. For example, males with gastric cancer suffer a worse prognosis than females; moreover, male sex was an independent poor prognostic risk factor [18]. On the other hand, women with bladder cancer suffer a worse prognosis and survival when compared to their male counterparts [19]. Thus, patient outcomes correlate differently with sex in different cancers. Consistent with this, analysis of cancer incidence using the Surveillance, Epidemiology, and End Results (SEER) database demonstrates significant differences between males and females in many cancers [20]. Despite these findings, the majority of studies in the cancer neuroscience field utilize only male mouse cancer models or combine data from males and females [21]. Thus, the current literature limits the understanding of sex as a biological factor with respect to tumor innervation and/or its influence on disease. Importantly, pain is one of the first symptoms leading patients to seek medical evaluation. While sex differences in cancer pain have been documented, reports have been mixed [22–25]. The extent of and type of nerves present within malignancies may explain some of this discrepancy. The oral cavity receives direct innervation from five cranial nerves (V, VII, IX, X and XII) making it one of the most densely innervated regions of the body [26]. Thus, to address the knowledge gap, we have used head and neck squamous cell carcinoma (HNSCC) as a model cancer in which to assess the influence of sex on tumor innervation, disease progression, treatment response, and outcomes. HNSCCs are cancers that arise in the oral cavity, the oropharynx, and the larynx. Twenty-five percent of cases are caused by infection with high-risk human papillomaviruses (HPV) and are referred to as HPV-positive (HPV+). The remaining cases are instead associated with the risk factors of smoking cigarettes and drinking alcohol and are referred to as HPV-negative (HPV-). While predominantly a male cancer [27], the incidence of HNSCC in females remains clinically important. Studies focused on sex differences in HNSCC have yielded mixed results [28–32]; whether cancer-associated nerves contribute to patient outcomes for this cancer remains unclear.

While local innervation of the tumor bed influences disease [15, 17, 33–35], the impact of these nerves extends far beyond the malignancy itself. Using a murine model of HPV - HNSCC, we have shown that these tumors are infiltrated by TRPV1-expressing nociceptor neurons that extend from the ipsilateral (tumor side) trigeminal ganglion [36]. When tracer mapped, these tumor-infiltrating nerves connect to specific regions in the brain [36, 37]. Neurons within this circuit are transcriptionally and functionally altered resulting in behavioral changes. Importantly, HNSCC patients suffer a high incidence of mental health decline [38–40]; this decline is maintained even in long-term cancer survivors [41]. Our behavioral studies in tumor-bearing mice suggest that tumor-infiltrating nerves, via their connection to the brain, may contribute to the mental health decline evident in cancer patients. Thus, it becomes important to define whether sex differences in behavior occur in tumor-bearing animals. We investigated the impact of oral malignancy on the activation status of central neurons and glia as such changes may influence behaviors. In addition, because cancer patients receive treatment (surgery, radiation, chemotherapy, immunotherapy, targeted therapy or their combinations), understanding how standard-of-care treatments impact tumor-infiltrating nerves as well as the neurons and glia in central projection areas, may provide a deeper understanding of the potential influences on behavior; here too, defining sex differences remains critical. Finally, given that metastatic disease is the predominant cause of cancer-associated death, we assessed the presence and extent of metastatic disease, its correlation with tumor innervation, and the influence of sex. Defining the influence of sex on these key aspects of oral cancer will expand the current understanding of sex as a biological factor in cancer.

## Materials and methods

### Cell lines

The *m*ouse *o*ral *c*arcinoma (MOC) 2–7 cell line (RRID: CVCL_ZD34) has been previously described [42]. Cells were maintained in Dulbecco’s Modified Eagle Medium (DMEM, Fisher, MT10017CV) with 10% fetal bovine serum (FBS, Hyclone, SH3039603) at 37 °C with 5% CO_2_. The mouse HPV16 E6, E7, Ras and luciferase expressing (mEERL95)(CVCL_B6JB) cell line is a derivative of the mEERL cell line (RRID: CVCL_B6J3). Both cell lines have been previously described [43, 44]. mEERL95 cells were cultured in DMEM/Ham’s F12 1:1 mixture with 5% FBS, GlutaMAX, 1x human keratinocyte growth supplement (ThermoFisher) and 1% penicillin/streptomycin at 37 °C with 5% CO_2_. MOC2-7 and mEERL95 cells grow in male and female C57BL/6 mice, were authenticated and tested free of mycoplasma.

The mouse melanoma cell line, YUMMER1.7 (CVCL_A2AX) has been previously described [45]. Briefly, this cell line is a derivative of the YUMM1.7 cell line following exposure to three rounds of UVB radiation (which increased the number of somatic mutations). YUMMER1.7 cells were cultured in DMEM/Ham’s F12 media supplemented with 10% FBS and 1% penicillin/streptomycin at 37 °C with 5% CO_2_.

### Animal studies

Male and female 6–7-week-old C57BL/6 mice were purchased from The Jackson Laboratory (catalogue #000664). Mice were acclimated to the facility for 2 weeks prior to the start of any study. Mice were ear-notched for identification.

### Tumor cell implantation

MOC2-7 and mEERL95 cells were grown and passaged at least twice in antibiotic-free media before implantation. Cells were washed twice with 1x phosphate-buffered saline (PBS), then trypsinized with 0.05% trypsin for 5 min. Trypsin (Corning, MT25051CI) was inactivated with PBS containing 2% FBS. Cells were centrifuged at 300x g for 5 min and diluted in pure DMEM to the appropriate cell concentration/volume for injection. For all head and neck cancer tumor injections, a cell concentration of 50,000 cells per 50 µl was used. Cells were kept on ice until injected.

YUMMER1.7 melanoma cells were similarly grown and passaged prior to tumor implantation. Females were injected with 2.5 × 10^6^ while males were injected with 1.5 × 10^6^ cells.

Mice were fully anesthetized with a cocktail consisting of 87.5 mg/kg Ketamine and 10 mg/kg Xylazine via intraperitoneal injection. Mice were placed in empty recovery cages on warmers kept at 37 °C and eye ointment was applied to prevent eyes from drying out. The cell solution was manually inverted to ensure an even distribution of cells, and the cells were drawn up into a 1 ml syringe. For oral tumors, once fully anesthetized, forceps were used to pull the lip away from the injection site and a 25-gauge needle was inserted, bevel-side up, and perpendicular into the back corner of the mouth into the cheek pouch (behind the whisker pad). Fifty microliters of the cell suspension were then slowly infused. Mice were returned to their home cages once fully awake. For melanoma tumors, mice were similarly anesthetized and injected intradermally in the back, behind the shoulder blades. Once anesthetized, the skin between the shoulder blades was tented and the needle (27-gauge, insulin needle) was inserted bevel-side up and the cells were slowly injected.

### Tumor growth

To measure tumors, mice were briefly anesthetized with isoflurane. Tumors were measured with digital calipers for length and width (mm). Tumor volume was calculated with the formula: Volume = ½ (length x width^2^). Tumor growth was limited to 1000mm^3^. Weight loss began after tumors surpassed 500mm^3^, during which time soft food was introduced on the cage floor to encourage eating and mitigate additional weight loss.

### RTX treatment

Resiniferatoxin (RTX), a naturally occurring compound from a cactus-type plant, acts as a potent analog of capsaicin (a TRPV1 agonist). RTX binds to TRPV1 inducing its hyperactivation and ultimately resulting in TRPV1 neuron ablation. As such, RTX-mediated chemoablation has been used clinically for pain relief [46]. To chemo-ablate TRPV1 neurons in mice, 4-week-old C57BL/6 mice were treated with three injections of RTX given 24 h apart. Because the first injection causes pain, animals were given 0.1 mg/kg of buprenorphine (given intraperitoneally) prior to the first RTX injection. Subsequent RTX injections are no longer painful and thus buprenorphine is not administered. RTX injections were given in the flank and consisted of 30 µg/kg on day 1, 70 µg/kg on day 2 and 100 µg/kg on day 3. Control animals received injections with vehicle (DMSO, Tween80, PBS). Following the last RTX injection, it takes four weeks for nerve ablation to occur. Ablation is confirmed with the tail flick test at 4 weeks.

### Tail flick test

Thirty minutes prior to testing, mice are acclimated to the testing room. A 52 °C water bath was used with a thermometer constantly monitoring the temperature so as to prevent any tissue damage from the water getting too hot. Following acclimation, each mouse was picked up using a clean paper towel and held such that its tail hung down outside of the paper towel. After the mouse relaxes in the tester’s hand, the tail is lowered into the water bath such that a quarter of the tail is submerged. The time for the mouse to flick its tail out of the water is recorded. If no response has occurred by 20 s, the tail is removed from the water so as to prevent tissue damage. A response latency time of greater than 10 s confirms denervation.

### Behavior assays

Mice were acclimated to being individually housed for two weeks prior to the start of behavior testing. During this time, mice were handled on at least three separate occasions by scruffing for 30 s, then held in an open palm for 30 s. This acclimated the mice to handling. In addition to the behavioral assays described below, mouse weight was monitored and recorded throughout the studies.

#### Nest building and burrowing

Nest building and burrowing are innate rodent behaviors exhibited equally by both sexes and are general indications of well-being. To assess nesting behavior, mice were individually housed and provided with a square nestlet (Ancare, NES3600) overnight. Mice shred the nestlet to build a nest. The following morning, the nests were scored on a scale of 0 to 4 by two independent scorers blinded to the conditions. Scoring of nests is standardized; a score of 0 indicates the mouse did not touch the nestlet at all, 1 indicates the mouse shredded less than 10% of the nestlet, a score of 2 indicates the mouse shredded greater than 50% of the nestlet but did not form a nest, 3 indicates the mouse shredded greater than 50% of the nestlet but the nest is less than half the body height of the mouse, 4 indicates the mouse shredded over 50% of the nestlet and the nest is greater than half its body height [47]. Mice were exposed to the cotton nestlet twice during their two-week single-housing acclimation period to ensure they were able to shred the nestlets. Three baseline tests were performed prior to tumor implantation. The average of the three baseline tests was utilized as the baseline score. Any mouse that did not average above a score of “3” was excluded from the data set (as a poor nester).

To assess burrowing behavior, mice were tasked with removing corncob bedding from a slightly elevated tube; they were given 30 min for this task. Mice were acclimated to the room for 20 min before starting the test. The weight of the bedding in the tube was recorded before and after the test [48]. Three baseline tests were assessed prior to tumor implantation, and their average was utilized as the baseline score. Both sexes were able to successfully remove the corncob bedding. The amount of bedding removed is used as the measure of burrowing.

#### Open field test

The open field test is designed to measure locomotor activity [49]. This test was performed in a dimly lit room to reduce stress induced by a bright light. Mice were acclimated to the room for 20 minutes prior to testing. Mice were placed in a box (16”x16”x13.5”) with a camera above the box to record the mouse’s movements using the AnyMaze software. Boxes were cleaned after each use.

#### Voluntary wheel running

Mice were acclimated to the running wheel for two weeks prior to testing. The two-week acclimation period also served to stabilize running performance. Running wheels remained in the cages for the duration of the study. The only time the wheel was removed from the cage was on nights when nesting performance was evaluated. Given that mice are nocturnal animals, only nighttime running from 8pm to 8am was evaluated. Running data were collected continuously in 1-minute bins as we have previously described [36, 50].

### Chemotherapy & radiation of oral tumors

Chemo- and radiotherapy were performed simultaneously, once per week for two or three weeks (on specified days post-tumor implantation). Mice were first anesthetized with a cocktail of 87.5 mg/kg Ketamine and 10 mg/kg Xylazine. Once anesthetized, mice were given an intraperitoneal cisplatin injection (5.28 mg/kg). Cisplatin was mixed with PBS and kept in the dark until injected. Control animals received vehicle only.

Radiotherapy treatment consisted of 8 Gy of radiation. Anesthetized mice were placed in individual lead-lined shielding devices with only the tumor exposed (facing up) through a hole in the device. Mice were held in place gently with medical tape. Lead tape was used to adjust the size of the hole depending on the size of the tumor to ensure that only the tumor was exposed to radiation. Anesthetized mice within their shielding devices were then carefully placed inside the irradiator (RS-2000, RadSource) and exposed to 8 Gy X-ray radiation. After irradiation, mice were injected subcutaneously with 100 µl saline to prevent dehydration. Untreated mice were given the same anesthesia and 100 µl saline required for radiation treatment. Mice were placed in recovery cages and kept at 37 °C until fully awake, after which they were returned to their home cages.

### Nerve tracing

Wheat Germ Agglutinin (WGA) conjugated to A568 (Invitrogen #W56133) was injected intra-tumorally as previously described [37]. Briefly, mice were anesthetized with the Ketamine/Xylazine cocktail described and a 10 µL Hamilton syringe with an attached 30 gauge needle loaded with 1% WGA. To expose the oral cavity, the lower lip of the mouse was pulled outward using forceps, and 2 µL of WGA was slowly injected (over the course of 10 min) into the tumor bulk, approximately halfway into the tumor and ensuring that the needle bevel was facing up. Once the volume was injected, the needle remained in place for 2 additional minutes to ensure that the tracer did not leak out. Mice were placed on a heating pad to recover and, once freely mobile, they were returned to their home cages for 5–7 days to allow retrograde transport of the tracer before euthanasia.

### Tissue collection & fixation

When euthanasia criteria were met, animals were euthanized with CO_2_, followed by cardiac perfusion with PBS and then 10% formalin. The tumor, lungs, lymph nodes, brain, and trigeminal ganglia were collected and fixed in 10% formalin for at least 24 h. Tissues were then transferred to PBS; brains were transferred to 30% sucrose. For studies that required live TGM neurons, animals were not fixed post-euthanasia.

### Immunofluorescent staining

Tumors were embedded in paraffin, sectioned (4 μm), and mounted onto glass slides. To dewax, slides were incubated in xylene for 5 min with agitation every 30 s, followed by a series of 1-minute washes in 100%, 90%, and 70% EtOH. Slides were briefly washed in H_2_O for 1 min before being transferred to pre-warmed antigen retrieval buffer (10mM sodium citrate, 0.05% Tween 20, pH 6.0) for 30 min in a 95 °C water bath and 20 min at room temperature (RT), after which they were washed in running water for 5 min. Slides were dried and a ring was drawn around each tissue section using an Immedge pen (Vector Labs). PBS was used to keep the tissue from drying out. Slides were blocked with blocking buffer (3% goat serum, 1% BSA, in PBS) containing 0.01% Tx-100 for 30 min at RT and then washed with PBS. Sudan Black (diluted 1:20 in 70% EtOH) was applied for up to 1 min, followed by PBS washes (3 × 5 min). Slides were incubated in primary antibody diluted in blocking buffer (1:500) in a humidified chamber overnight at 4 °C. Slides were washed 3x for 5 min with PBS and incubated in secondary antibody, diluted in blocking buffer (1:500) in a humidified chamber in the dark for 1 h at RT. Slides were washed 3 × 5 min with PBS. For single antibody staining, slides were then counterstained with Hoechst (1:10,000 in PBS) and washed 2 × 5 min in PBS. For dual staining with antibodies of the same host species, fluorophore-conjugated antibody diluted in blocking buffer (1:500) was applied, and slides incubated in a humidified chamber overnight in the dark at 4 °C, followed by Hoechst incubation and washes. Glass coverslips were mounted with Fluoromount-G Mounting Medium (Invitrogen, #00-4958-02). Staining was analyzed using a confocal microscope (Nikon A1 TIRF). For each tissue, three to four images of representative regions were taken on the confocal microscope at 20x magnification.

### Multielectrode array

Extracellular recordings of tissue sections were collected using an Axion Maestero Edge (Axion Biosystems) recording system with 64 extracellular recording electrodes (50 μm diameter) arranged in an 8 × 8 grid (300 μm spacing, 2.1 mm x 2.1 mm recording area) with integrated ground electrodes per well on a 6-well multielectrode array (MEA). All voltage data were filtered using dual 200 Hz (high pass) and 3 kHz (low pass) filters. Waveforms were collected with the Axion AxIS Navigator (Version 3.6.2.2) with detection criterion of > 6 SDs above background signals with 12.5 kHz sampling frequency. Mouse tumors were quickly dissected and immediately sectioned using a scalpel; *n* ≥ 3 slices were analyzed at a minimum (larger tumors allowing for a larger number of slices). Sections were randomly placed in different wells distributed over the same MEA plates. Sections were kept in contact with electrodes using a tissue anchor and were maintained under oxygenated (95% O_2_, 5% CO_2_) artificial cerebrospinal fluid (119 mM NaCl, 2.5 mM KCl, 1 mM NaH2PO4, 26.2 mM NaHCO3, 11 mM glucose, 1.3 mM MgSO4, and 1.5 mM CaCl2), at 37 °C and recordings obtained over 2-min intervals. Analysis considered tumor sections demonstrating electrical activity. Sections in which none of the electrodes harbored activity were not included in the analysis. ‘Active’ sections were defined as having active electrodes at 5 spikes/minute; electrode bursts were defined as at least five consecutive spikes with interspike intervals less than 100 ms. MEA recordings from a total of 27 male untreated slices, 24 male treated slices, 16 female untreated slices, 18 female treated slices were analyzed.

### Ca^2+^ imaging of dissociated TGM neurons

Trigeminal ganglia were harvested and enzyme-digested in papain, and then collagenase II/dispase at 37 °C for 20 min. After washing and trituration, cells were plated onto a thin layer of Matrigel in glass-bottom dishes and cultured with Ham's-F12 supplemented with 10% FBS. The cells were maintained in an incubator (5% CO_2_, 37 °C) for 24 h before they were used for calcium imaging experiments. Neurons were incubated with the calcium indicator, Fluo-4AM, at 37 °C for 20 min followed by another 20 min at RT to allow de-esterification of the indicator. The cells were then washed, and Live Cell Imaging Solution (Thermo-Fisher) with 20 mM glucose was added. Calcium imaging was conducted at RT. Changes in intracellular Ca^2+^ were measured using a Nikon scanning confocal microscope with a 10x objective. Fluo-4AM was excited at 488 nm using an argon laser with intensity attenuated to 1%. The fluorescence images were acquired in the confocal frame (1024 × 1024 pixels) scan mode. After 1 min of baseline measurement, capsaicin (300 nM final concentration) was added. Ca^2+^ images were recorded before, during, and after capsaicin application. Image acquisition and analysis were achieved using NIS-Elements imaging software. Calcium responses were analyzed only for neurons that subsequently responded to ionomycin (10 µM) to ensure neuronal health.

### Methods for quantifying neuronal and glial activation in the brain

Neuronal and glial activation within the brains of tumor-bearing mice was assessed in the absence of any specific stimulus to elicit activation. Here, we are assessing if the presence of a peripheral oral tumor is sufficient to trigger neuronal/glial activation in the brain. Mice were transcardially perfused with PBS, followed by 4% paraformaldehyde in PBS. Brains were extracted and postfixed in 4% paraformaldehyde for 24 h at 4 °C. The tissue was then sectioned into 40 μm sagittal slices from the ipsilateral or contralateral hemisphere using a vibratome and transferred to 24-well plates loaded with 0.1 M PBS (pH 7.4) 0.03% sodium azide. All sections were blocked with 10% normal goat serum in PBS containing 0.03% Triton X-100 for 30 min. Sections were then incubated with anti-Fos antibody (rabbit, 1:10000, Cell Signaling Technology, #2250) or anti-FosB antibody (mouse, 1:5000, Abcam, Ab11959) for 72 h. Other sections were incubated with Anti-GFAP (rabbit 1:1000, Agilent Dako, Z0334) or Anti-Iba1 (rabbit ,1:100, Abcam, ab178847) antibody. Following washes in 0.1 M PBS, sections were incubated in secondary antibodies for 3 h. (Alexa568 anti-rabbit, 1:500, Thermo Fisher, A-11011 or Alexa488 anti-mouse, 1:500, Thermo Fisher, A-11001). Following additional washes, sections were mounted onto glass slides and counterstained with DAPI (Thermo Fisher, #62247) prior to imaging.

### Lung metastasis quantification

Animals were perfused, their lungs harvested, embedded in paraffin, and cut in 4 μm sections. To assess metastatic lesions, three sections/mouse were immunohistochemically stained for cytokeratin (a marker used to identify metastases). These sections were each separated by 20 μm enabling assessment of metastatic lesions through a depth of the lungs. Stained slides were digitally scanned at 20x magnification using an Aperio Versa Scanner. The number and area of cytokeratin-positive metastases were quantified using Aperio Image Scope. Briefly, each metastatic lesion was circumscribed throughout each section. Any positively stained region that comprised > 10 cells was counted. This information was quantified as the total number of metastatic lesions as well as total metastatic lesion area per lung.

### ImageJ analysis

The number of Fos-labeled nuclei within the region of interest (ROI) was counted on 2 D images acquired using a laser-scanning confocal microscope (Nikon A1R) and verified as positive if the signal filled the nucleus and stood out clearly compared to surrounding tissue. Quantification of Fos-labeled cells was performed using digital thresholding of Fos-immunoreactive nuclei. For each animal, 3 sections were selected at 120 μm intervals. ImageJ cell counter (v. 1.52) was used to quantify Fos+ nuclei per region / section. To quantify glial cells, which have many overlapping projections, photomicrographs were converted to 8-bit and thresholded. The area fraction for photomicrographs were averaged to obtain a single value per mouse.

For quantification of tumor innervation, Z-stacks collected on the confocal microscope were opened as a hyperstack. The target channel was isolated, and the z-stack was compressed to a maximum intensity image. The lowest threshold was set and used for all images. The total area of the positive staining (nerves) was measured. If nerve bundles were present, they were traced, and an ROI was created. The area of the nerve bundle was measured and subtracted from the total area.

### Statistical analysis

GraphPad Prism 10 and/or R version 4.4.0. were utilized for all statistical analyses. R packages used included rstatix, Ime4 and emmeans. The specific statistical test used for each experiment is noted in the figure legend and explained below.

Figure 1. Data were analyzed by 2-way repeated measures ANOVA.

Figure 2. Data were analyzed by Student’s t-test.

Figure 3. Qualitative data.

Figure 4. Qualitative data.

Figure 5. Burrowing (Panel A) and open field distance (Panel B) were analyzed using mixed-effects models due to missing data points. Time in zones (Panel C) was analyzed by 2-way repeated measures ANOVA. Post-hoc comparisons used Tukey’s test.

Figure 6. Data were analyzed by three-way repeated measures ANOVA; Mauchly’s test indicated violation of sphericity; Greenhouse-Geisser corrections were applied to degrees of freedom, with post-hoc Tukey’s test.

Figure 7. Nesting scores were analyzed using a three-way repeated measures ANOVA with Days as a within-subjects factor and Treatment and Sex as between-subjects factors. Mauchly’s test indicated violation of sphericity (*p* < 0.001); therefore, Greenhouse-Geisser corrections were applied to degrees of freedom. Significant interactions were followed by post-hoc pairwise comparisons with Tukey’s HSD adjustment for multiple comparisons. Analyses were performed using R version 4.4.0 with the rstatix, lme4, and emmeans packages.

Figure 8. Statistical analysis by 2-way ANOVA.

Figure 9. Data for panel A and B were analyzed by 2-way ANOVA with Tukey’s multiple comparisons test. Data for panel C were analyzed by 3-way ANOVA with multiple comparisons.

Figure 10. Data were analyzed by 2-way ANOVA with post-hoc Tukey’s test.

Figure 11. Data were analyzed by 2-way ANOVA with post hoc Tukey’s test.

Figure 12. Data were analyzed by 2-way ANOVA with multiple comparisons.

Figure 13. Statistical analysis by 2-way ANOVA.

## Results

**Fig. 1:**
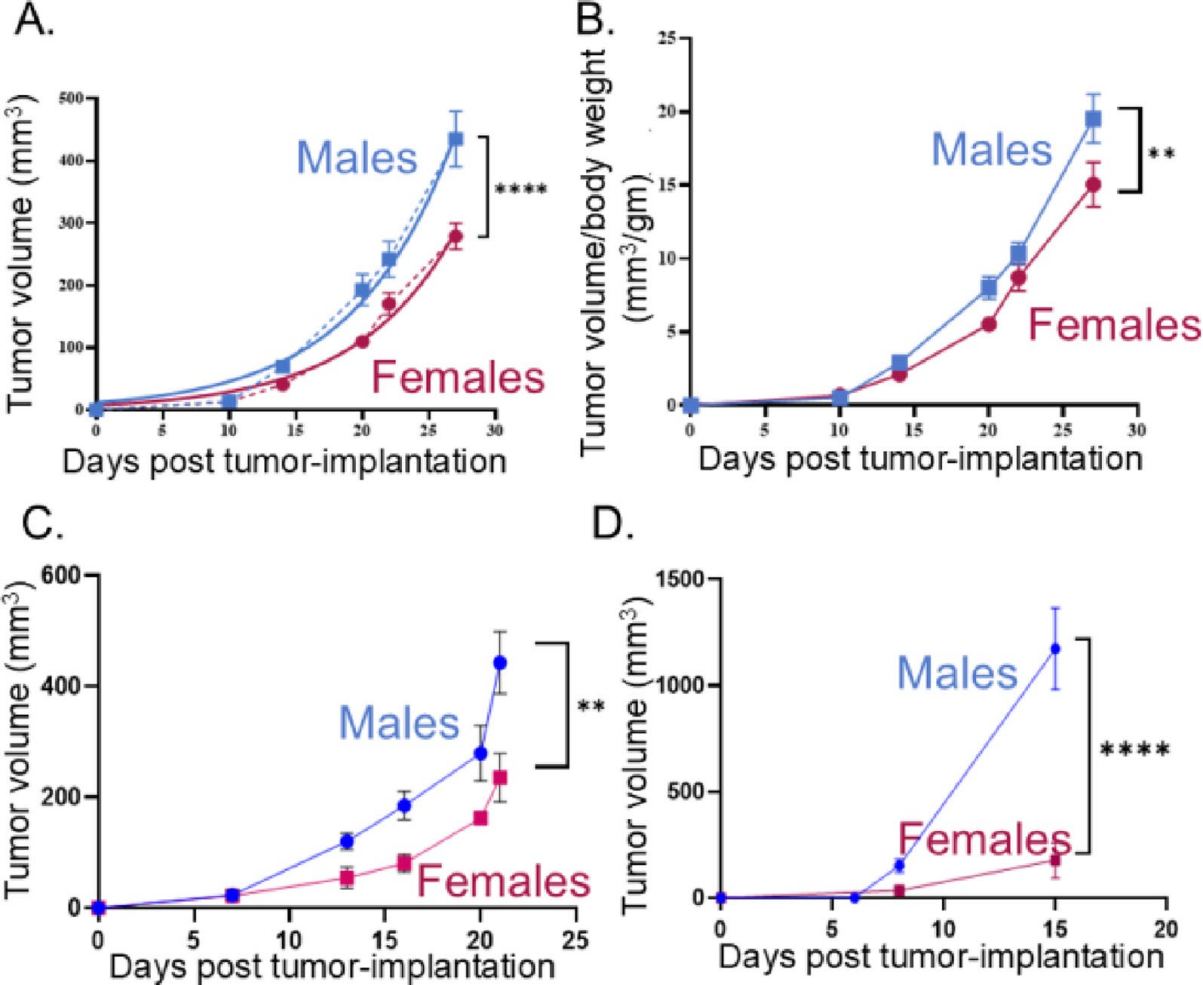
Tumors grow slower in females. **A**) MOC2-7 tumor volume plotted as a function of time. Male (blue), female (pink); *n* = 10 mice/group. Tumor growth plotted (dotted lines) with non-linear exponential growth curve (solid lines). **B**) Tumor volume was normalized to body weight and plotted as a ratio. The experiment was repeated twice by two different investigators with similar results. mEERL95 (**C**) and YUMMER1.7 (**D**) tumor growth plotted as a function of time. Male (blue), female (pink); *n* = 5 mice/group for each tumor type. Statistical analysis by 2-way repeated measures ANOVA for A-C. **, *p* < 0.05; ****, *p* < 0.0001. Statistical analysis by mixed effects model for D. ****, *p* < 0.0001. Error bars, SEM

### Tumors grow slower in female mice

To assess if there are sex differences in tumor growth for oral cancer, MOC2-7 cells were orthotopically implanted (oral cavity) into 8–9-week-old male and female C57BL/6 mice (*n* = 10 per group). These oral tumors grew significantly slower in female mice as compared to their male counterparts (2-way RM-ANOVA; Days: F(1.209, 19.34) = 207.5, *p* < 0.0001; Sex: F (1, 16) = 9.553, *p* = 0.0070; Days×Sex: F(1.209, 19.34) = 9.960, *p* = 0.0035; Fig. 1A; Supplemental Figs. 1A, B). To determine if the smaller tumor sizes merely reflected the smaller body size of females, tumor volume was normalized to body weight at each time point. When normalized to body weight, this sex difference persisted (Fig. 1B). To assess whether this sex difference was unique to MOC2-7 cells or a more generalized phenomenon, C57BL/6 mice (*n* = 5 mice/sex) were orthotopically implanted (oral cavity) with mEERL95 cells, a murine model of human papillomavirus-induced head and neck cancer [46]. Similar to MOC2-7 tumors, mEERL95 tumors exhibited significantly slower growth in females (2-way RM-ANOVA; Days: F(1.768, 12.38) = 47.93, *p* < 0.0001; Sex: F (1, 7) = 11.27, *p* = 0.0121; Fig. 1C). To assess whether the implantation site contributed to this sex difference, male and female C57BL/6 mice (*n* = 5 mice/sex) were subdermally implanted with a murine melanoma cell line, YUMMER1.7, in the dorsal skin between the shoulder blades. The YUMMER1.7 tumors grow transiently and regress due to a robust immune response; this can be overcome by implanting more cells [45]. YUMMER1.7 cells were derived from the YUMM1.7 cell line, which was generated from a male mouse [51]. As such, sex-specific immune responses explain YUMMER1.7 tumor rejection in females [52]. A similar immune effect leading to smaller tumors in females has also been documented for B16-F10, another melanoma model [53]. Thus, to overcome this rejection, we implanted females with more cells (2.5 × 10⁶/mouse) as compared to males (1.5 × 10⁶/mouse). Despite this, tumors grew significantly slower in females (Mixed-Effects model; Days: F(1.108, 33.97) = 21.44, *p* < 0.0001; Sex: F (1, 92) = 19.92, *p* < 0.0001; Fig. 1D).

### Tumors in female mice are more densely innervated

**Fig. 2:**
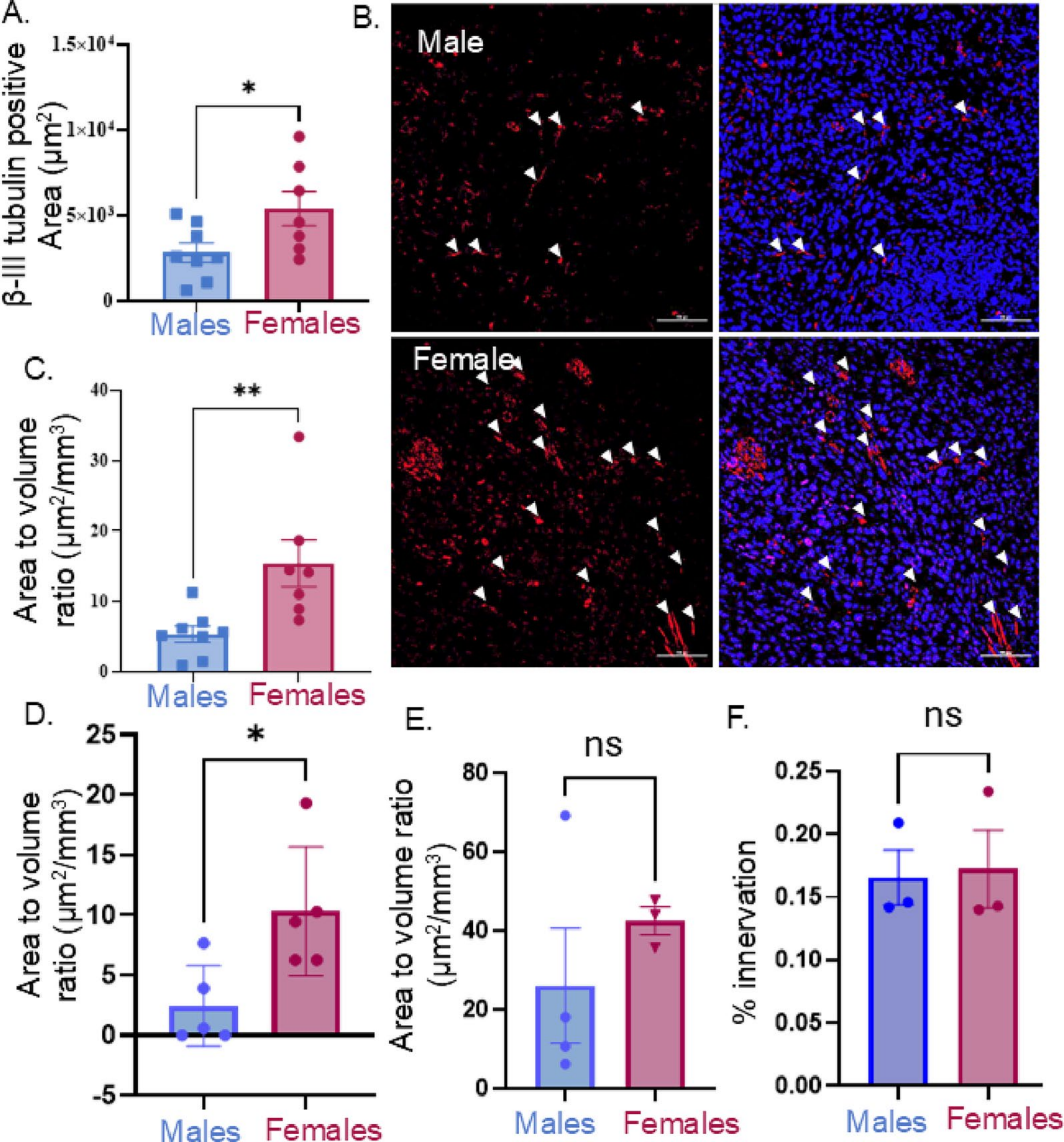
Dense tumor innervation in female mice. **A**) Confocal images MOC2-7 (HPV-) tumors were quantified for innervation using ImageJ software for the total area (mm^2^) of positive β-III tubulin staining. Average innervation area was calculated from 3–4 images per tumor (*n* = 7–8 tumors per group). **B**) Representative photomicrographs of β-III tubulin-stained axons (red), indicated with white arrows. Hoechst counterstain (blue). Scale bar, 100 μm. **C**) Average nerve area (mm^2^) of MOC2-7 tumors normalized to tumor volume (mm^3^). Quantification of positive β-III tubulin staining area to tumor volume ratio in oral mEERL95 (**D**) and YUMMER1.7 (**E**) tumors. **F**) Oral tissue from a non-tumor male and female mice was immunostained for β-III tubulin and innervation area quantified. Statistical analysis by Student’s t-test. *, *p* < 0.05; **, *p* < 0.001, ns, not significant

Our previously published studies have focused on single axons that infiltrate peripheral malignancies [11, 14]. Nerve bundles can sometimes also be found within tumors; these axons are surrounded by layers of connective tissue (endoneurium, perineurium and epineurium) which can be breached by tumor cells in a process known as perineural invasion (PNI) [54, 55]. Patients with evidence of PNI have a worse prognosis than those without [54]. However, our current study is not focused on PNI thus, to avoid any confusion, nerve bundles were not included in the quantification of tumor innervation. Our previous study assessing tumor innervation was performed using only male mice [17]. Thus, to compare the innervation density between male and female tumors, MOC2-7 tumors were implanted in the oral cavities of male and female mice (*n* = 10 mice/group). Tumors were harvested 31 days post-tumor implantation, and immunofluorescently stained for β-III tubulin, a pan neuronal marker. Given that MOC2-7 tumors grow slower in females, we were surprised to find that these tumors were significantly more innervated as compared to their male counterparts (Fig. 2A, B). When innervation area was normalized to tumor volume, female tumors remained significantly more innervated than male tumors (Fig. 2C). Likewise, the innervation area to tumor volume ratio of mEERL95 and YUMMER1.7 tumors was also significantly greater in females compared to males (Fig. 2D, E). Innervation of the female mouse tongue is more dense than that of the male [56]. We wondered if a similar sex difference accounts for the increased tumor innervation of oral tumors. Thus, the oral tissue of non-tumor male and female mice was fixed, immunostained for β-III tubulin and innervation quantified. We found no significant difference in innervation between males and females (Fig. 2F). These findings suggest that sex, not the site of tumor implantation, contributes to tumor growth and innervation differences. These findings were surprising given that previous studies from our group as well as others, show that faster growing tumors are more densely innervated [16, 17, 57]. Given the similar findings in these three tumor types, additional studies were performed only with MOC2-7 oral tumors.

**Fig. 3:**
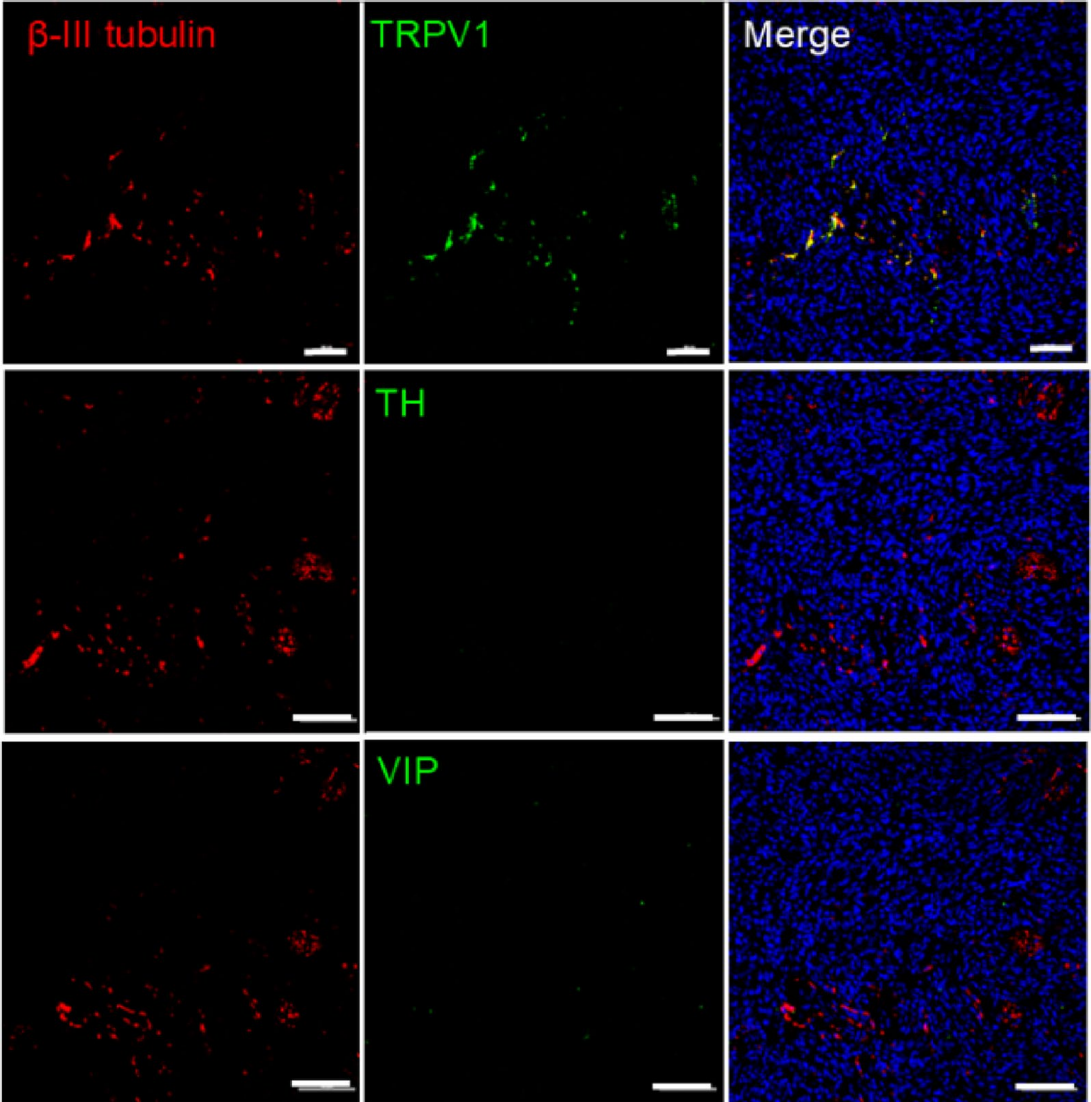
Sensory nerves infiltrate MOC2-7 tumors in females. Oral MOC2-7 tumors from female mice (*n* = 3) were immunofluorescently co-stained for β-III tubulin (red) and either TRPV1, TH, or VIP (green). Hoechst was used as a nuclear counterstain (blue). Stained tumors were analyzed by confocal microscopy. Three to four images/tumor/mouse were taken at 20x magnification and analyzed for axons. Scale bar, 100 μm

We have previously determined that MOC2-7 tumors in male mice are innervated with TRPV1-expressing sensory nerves [37]. To determine the type of nerves infiltrating MOC2-7 tumors in females, tumors were immunofluorescently stained for β-III tubulin as well as TRPV1 (sensory marker), tyrosine hydroxylase (TH, sympathetic marker) and vasoactive intestinal polypeptide (VIP, parasympathetic marker). This qualitative analysis suggests that, like their male counterparts, female MOC2-7 tumors are innervated by TRPV1-expressing sensory nerves (Fig. 3).

### The tumor-brain circuit does not differ between males and females

**Fig. 4:**
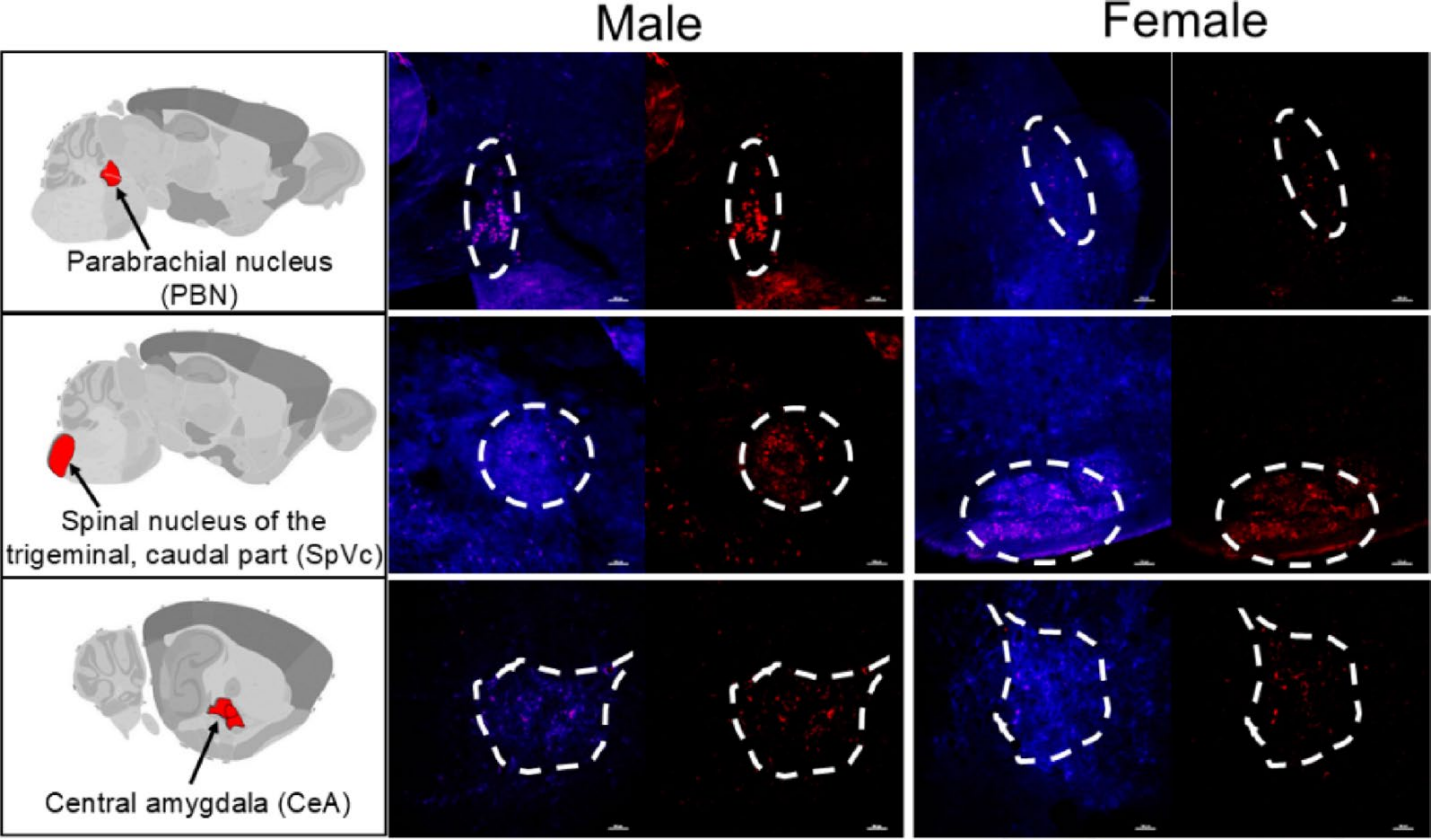
No sex differences in the tumor-brain circuit. Positive WGA neural tracer (red) found in the PBN, SpVc, and CeA in both male and female tumor-bearing animals (*n* = 4). 10x magnification, scale = 100 μm. Diagrams from Allen Reference Atlas – Mouse Brain, atlas.brain-map.org

Neural tracers are fluorescently labeled molecules or viruses that are taken up by nerve terminals. Depending on the tracer, they are transported retrogradely or anterogradely within the neuron. Several tracers have trans-synaptic capabilities, enabling the mapping of neural circuits [58, 59]. This approach has been extensively utilized to map brain circuits in rodents [60–63]. Using intra-tumoral injection of nerve tracer, we previously mapped the circuitry of tumor-infiltrating nerves in oral tumors and found that they connect to a pre-existing circuit that extends into the brain [36]. Specifically, oral MOC2-7 tumors in male mice are innervated by nerves that emerge from the V3 branch of the ipsilateral trigeminal (TGM) ganglion and connect with the spinal nucleus of the trigeminal (SpVc), the parabrachial nucleus (PBN), and the central amygdala (CeA) [36]. To determine if this tumor-brain circuit differs with sex, MOC2-7 oral tumor-bearing male and female mice (*n* = 4 per sex) were intra-tumorally injected with tracer and euthanized 5–7 days later (allowing sufficient time for tracer transport to the brain). TGM ganglia and brains were harvested and sections analyzed by confocal microscopy for the presence of tracer. Like male mice, axons within oral MOC2-7 tumors in females mapped to the ipsilateral TGM, and then the SpVc, PBN, and CeA (Fig. 4). These tracing data indicate that the anatomical tumor-to-brain circuit appears conserved between male and female mice; however, whether the functional strength of this connection differs by sex is not addressed by this approach.

### Tumor-associated changes in behavior

**Fig. 5:**
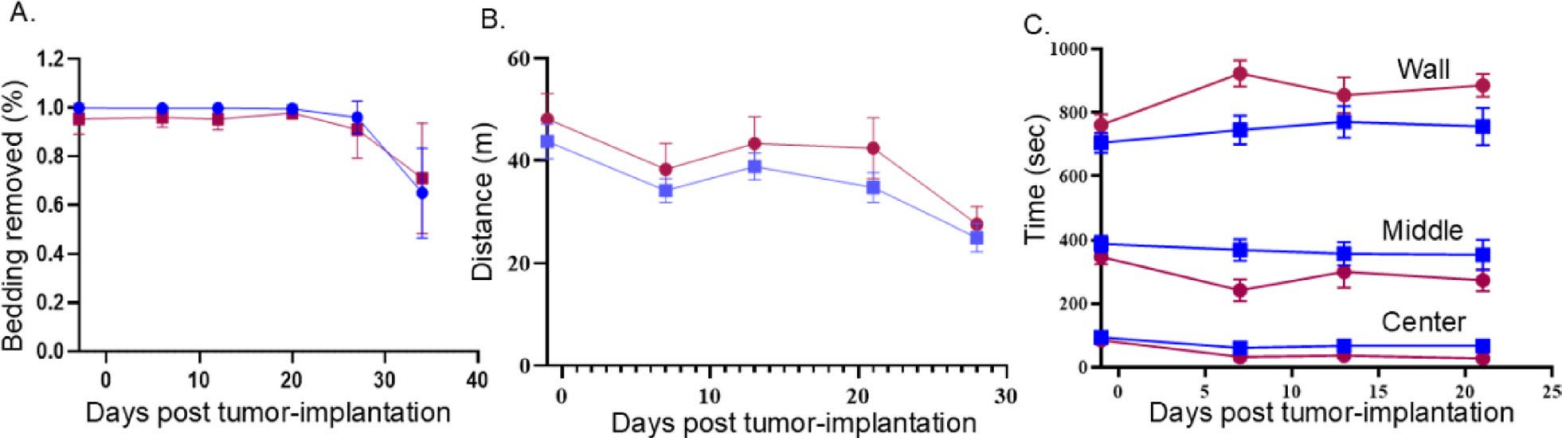
Cancer-associated behavioral declines. **A**) Burrowing behavior assessed as amount of bedding removed from burrowing tubes as a function of time. **B**) Graph of the total distance traveled in the open field test over time. **C**) Graph of the time spent in wall, middle, and center zones in the open field test. Blue, males; red, females. Statistical analysis by mixed effects model for panels A and B and by repeated measures 2-way ANOVA;. *n* = 8–10 mice/group. Data are mean ± SEM

While inflammatory cytokines associated with peripheral malignancies certainly influence the central nervous system and correlate with cancer-associated behavioral alterations [64–67], our identification of a neural circuit linking peripheral oral tumors with projections into the CNS suggests a more direct interaction may also contribute to these behavioral changes [36]. The increased tumor innervation evident in female oral MOC2-7 malignancies prompted us to ask whether cancer-associated behavioral changes differ between the sexes. Two behaviors were assessed: burrowing and open field. Burrowing is an innate behavior exhibited equally by male and female mice and serves as a measure of well-being [47, 48]. The open field test is used as a general measure of exploratory behavior [49]. Prior to tumor implantation, mice underwent behavioral testing to obtain baseline measures. Following tumor implantation, behavioral assessments were completed weekly. For burrowing, a mixed-effects model revealed a significant effect of time (*p* < 0.0001), with no significant effect of sex (*p* = 0.31) or time × sex interaction (*p* = 0.35) (Fig. 5A).

In the open field test (mixed-effects model), females traveled significantly greater distances than males (Sex: F (1, 65) = 4.726, *p* = 0.033), and both sexes showed declines over time (Time: F (6, 77) = 5.879, *p* < 0.0001). However, the time × sex interaction was not significant (F (6, 65) = 0.113, *p* = 0.995), indicating that the temporal pattern of decline was similar between sexes (Fig. 5B). Analysis of time spent in different zones (2-way RM-ANOVA) revealed significant sex differences in the wall zone (F (1, 21) = 5.86, *p* = 0.024), middle zone (F (1, 20) = 4.38, *p* = 0.049), and center zone (F (1, 21) = 4.94, *p* = 0.037). Time and time × sex interactions were not significant for any zone (all *p* > 0.05; Fig. 5C). Individual data points for each mouse can be found in Supplemental Fig. 3A, B. These data show that sex did not influence burrowing behavior but did influence some aspects of the open field test (total distance traveled and time in the wall, middle, and center zones).

### Chemoradiation treatment partially restores nesting behavior

**Fig. 6:**
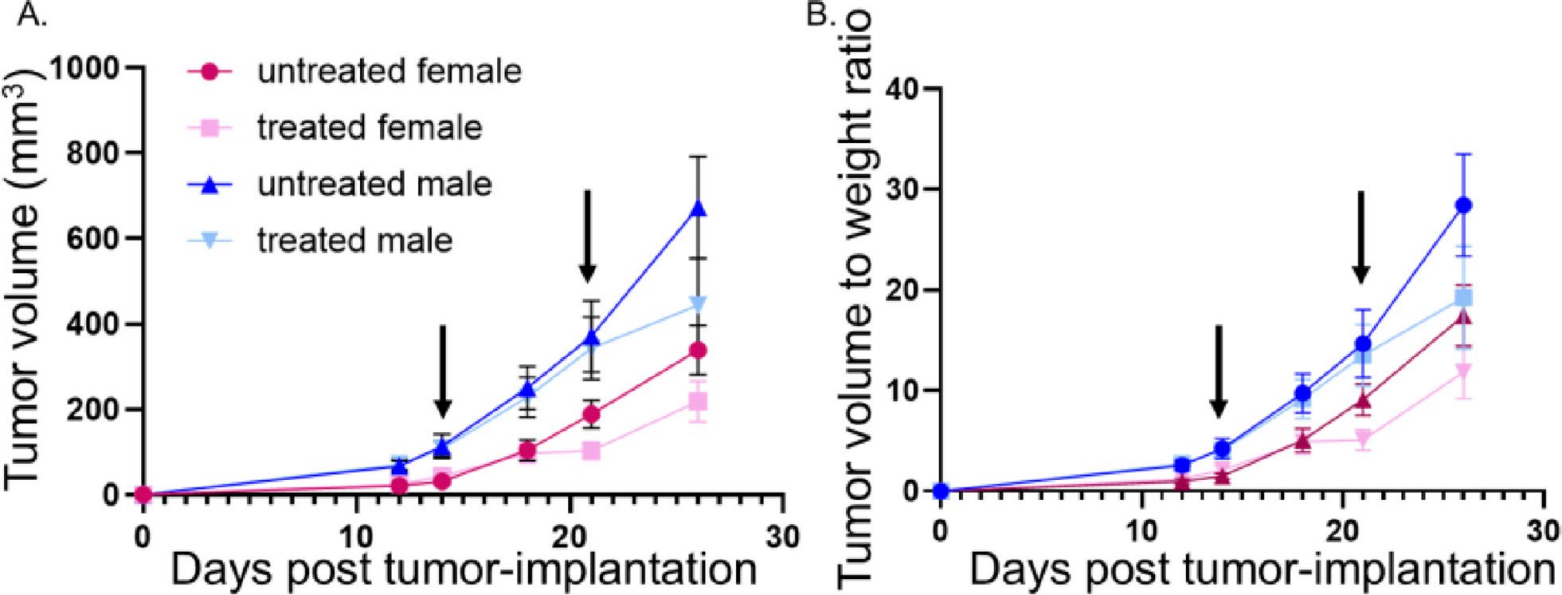
Tumor growth curves of untreated and treated mice. **A**) Growth of MOC2-7 tumors in untreated and treated males and females. Volume data were analyzed using three-way repeated measures ANOVA with Days, Treatment, and Sex. **B**) Graph of tumor volume/weight ratio for the same animals as in panel A. Volume/weight ratio data were analyzed using three-way repeated measures ANOVA with Days, Treatment, and Sex. Arrows indicate treatment (vehicle or cisplatin/radiation) days. *n* = 8 males/group; *n* = 12 females/group. Experiment repeated two times with similar results. Error bars, SEM

Given that cancer patients receive therapy, it is important to determine the influence of standard-of-care treatment on tumor growth and behavior. Thus, a cohort of mice was orally implanted with MOC2-7 tumors: untreated males (*n* = 8), treated males (*n* = 8), untreated females (*n* = 12), and treated females (*n* = 12). Treatment commenced on day 14 post-tumor implantation and consisted of cisplatin (intraperitoneal injection, 5.28 mg/kg) and radiation therapy (8 Gy) once a week for two consecutive weeks. Untreated mice received vehicle and were similarly anesthetized but not irradiated. A three-way repeated measures ANOVA (Treatment × Sex × Days) revealed significant main effects of Days (F(1.35,48.49) = 93.09, *p* < 0.001, Greenhouse-Geisser corrected) and Sex (F (1, 36) = 17.68, *p* < 0.001), with no main effect of Treatment (F (1, 36) = 1.81, *p* = 0.187). Significant Treatment × Days (F(1.35,48.49) = 4.68, *p* = 0.025) and Sex × Days (F(1.35,48.49) = 10.78, *p* < 0.001) interactions were observed, indicating that both treatment and sex effects changed over time. Post-hoc pairwise comparisons (Tukey-adjusted) revealed that treated animals exhibited significantly smaller volumes than untreated controls at Day 26 (*p* < 0.001), with no differences at earlier timepoints. Additionally, females showed significantly smaller volumes than males at Days 18, 21, and 26 (all *p* < 0.001; Fig. 6A). When normalized to body weight, the pattern remained consistent (Fig. 6B). Three-way RM-ANOVA for volume-to-body weight ratio revealed significant main effects of Days (F(1.32,47.55) = 87.68, *p* < 0.001) and Sex (F (1, 36) = 9.55, *p* = 0.004), with no main effect of Treatment (F (1, 36) = 1.57, *p* = 0.218). Significant Treatment × Days (F(1.32,47.55) = 3.89, *p* = 0.044) and Sex × Days (F(1.32,47.55) = 5.24, *p* = 0.018) interactions were observed. Post-hoc comparisons showed treated animals had significantly lower ratios than untreated controls at Day 26 (*p* < 0.001), with females exhibiting significantly lower ratios than males at Days 18, 21, and 26 (all *p* ≤ 0.022). When a second cohort was treated at an earlier timepoint for an additional week, these sex differences persisted (Supplemental Fig. 4A, B).

**Fig. 7:**
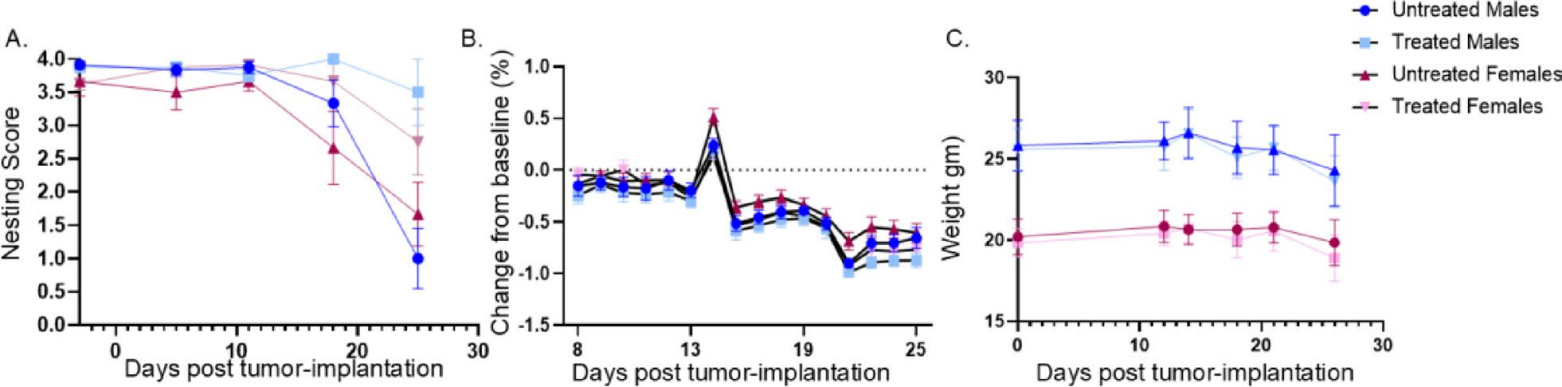
Impacts of cisplatin/radiation treatment on cancer-associated behaviors. **A**) Nesting scores, **B**) running wheel performance as % change from baseline, and **C**) body weight in MOC2-7 oral tumor-bearing mice. *n* = 12 females/group; *n* = 8 males/group. Data are mean ± SEM. Three-way repeated measures ANOVA for Panels A and B; mixed-effects model for Panel C. See Results for statistics. **p < 0.01; ***p < 0.001

We have previously published that nesting and running wheel performance declined with tumor progression and that commonly used analgesics (carprofen and buprenorphine) significantly attenuated this decline [36]. To determine whether standard-of-care cisplatin/radiation treatment similarly affects these behavioral outcomes, mice were singly housed, subjected to baseline behavior testing prior to tumor implantation and then tested repeatedly for the duration of the experiment. Nesting and wheel-running performance were assessed.

A three-way repeated measures ANOVA (Treatment × Sex × Days) revealed significant main effect of Treatment (F (1, 37) = 15.35, *p* < 0.001) and Days (F(2.11,78.24) = 22.55, *p* < 0.001,), with no main effect of Sex (F (1, 37) = 1.81, *p* = 0.187) on nesting performance (Fig. 7A). Importantly, a significant Treatment × Days interaction was observed (F(2.11,78.24) = 7.25, *p* = 0.001), indicating that treatment effects changed over time. Post-hoc pairwise comparisons (Tukey-adjusted) revealed no treatment differences at baseline (Day − 3, *p* = 0.887) or early timepoints (Days 7 and 14, *p* > 0.4). Treatment effects emerged at Day 19 (mean difference = 1.79, *p* < 0.001) and were sustained through Day 27 (mean difference = 1.79, *p* < 0.001), with treated animals exhibiting significantly higher nesting scores than untreated controls. Running performance was analyzed as percent from baseline (Fig. 7B). A three-way repeated measures ANOVA revealed a significant main effect of Days (F(5.214, 182.5) = 122.0, *p* < 0.001), with no main effects of Treatment (F (1, 35) = 2.769, *p* = 0.105) or Sex (F (1, 35) = 3.747, *p* = 0.061). No significant interactions were observed between Days and Treatment (F(5.214, 182.5) = 0.5407, *p* = 0.7572 ), Days and Sex (F(5.214, 182.5) = 1.879, *p* = 0.097), or Days × Treatment × Sex (F(5.214, 182.5) = 1.269, *p* = 0.278). These data indicate that voluntary running activity declined over time independent of treatment or sex. (Fig. 7B). Body weight was monitored throughout the experiment (Fig. 7C). As expected, females are smaller than males. A mixed-effects model revealed significant main effects of Days (F(3.326, 106.0) = 46.64, *p* < 0.0001) and Treatment (F (1, 37) = 177.9, *p* < 0.0001), with no main effect of Sex (F (1, 37) = 0.644, *p* = 0.428). A significant Days × Treatment interaction was observed (F(3.326, 106.0) = 4.148, *p* = 0.006), whereas interactions involving Sex were not significant. These data indicate that body weight changed over time in a treatment-dependent manner, with no effect of sex.

### Cisplatin/radiation treatment increases tumor innervation

**Fig. 8:**
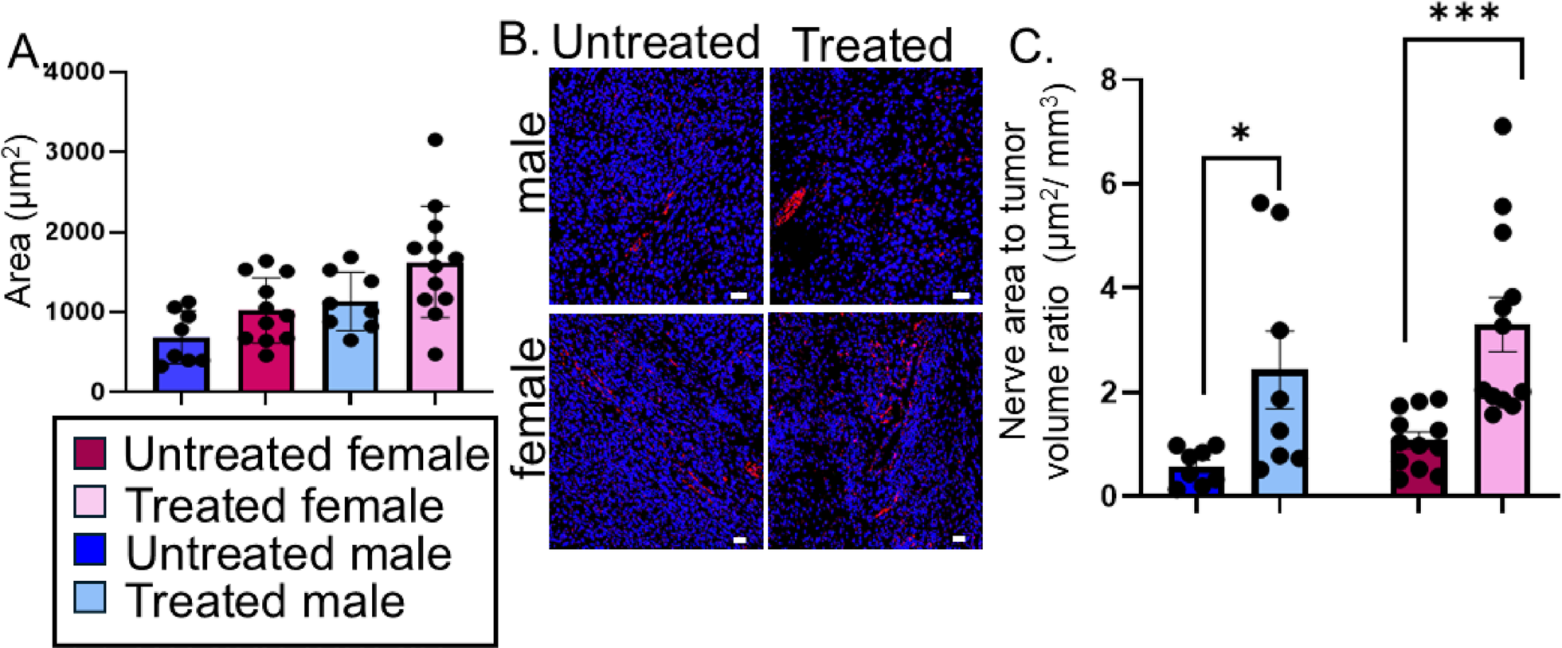
Cisplatin/radiation treatment increases tumor innervation. **A**) Quantification of the total area of positive β-III tubulin staining in tumors from the indicated groups. Confocal images (3–4 per tumor) were taken, and the area of β-III tubulin positive axons quantified. Statistical analysis by 2-way ANOVA with Tukey’s post hoc test. There were significant main effects of Treatment (F = 13.05, *p* = 0.000918) and Sex (F = 5.80, *p* = 0.0213) on the area of tumor innervation. **B**) Representative photomicrographs of axons stained with β-III tubulin (red). Nuclear counterstain (blue). 20x magnification, scale = 100 μm. **C**) Average nerve area normalized to tumor volume and values log transformed. Analysis by two-way ANOVA. There was a main effect of treatment, F (1, 36) = 20.20, *p* < 0.0001. Post-hoc Tukey’s test confirmed the treatment efficacy in males (*, *p* < 0.05) and females (***, *p* < 0.005). Error bars, SD

Our previous findings suggest that the tumor-brain circuit promotes neuronal activation in brain projection regions [36], and that chemoradiotherapy partially restores nesting performance. We therefore hypothesized that cisplatin/radiation treatment compromised the circuit’s integrity, thereby attenuating cancer-induced neuronal changes in the brain and, thus, behavioral alterations. To test this hypothesis, we first examined the impact of cisplatin/radiation treatment on local tumor innervation. Tumors from all groups were harvested and sections immunofluorescently stained for the pan-neuronal marker β-III tubulin. Staining quantification consisted of three images/tumor and included axons while excluding axon bundles (resident nerves). The total area of β-III tubulin positive staining was compared between the groups. To our surprise, we found that treatment increased innervation area and that females exhibit higher innervation area than males (Fig. 8A, B). Specifically, our analysis indicates that innervation area differs between treated and untreated groups when averaged across sexes and also differs between males and females when averaged across treatment conditions. The Treatment × Sex interaction was not significant (F = 0.415, *p* = 0.524), suggesting that the effect of treatment does not statistically depend on sex. Tukey post hoc comparisons for the main effects showed that untreated samples had significantly lower innervation area than treated samples (difference = − 572.8, adjusted *p* = 0.00092). Additionally, males had significantly lower innervation area than females (difference = − 389.7, adjusted *p* = 0.0213). Although the overall interaction term was not significant, pairwise comparisons across Treatment × Sex groups provide descriptive context. Specifically, untreated females had significantly lower innervation area than treated females (adjusted *p* = 0.014), and untreated males differed significantly from treated females (adjusted *p* = 0.0012). We normalized innervation density to tumor volume. This did not fit the model so the values were log transformed and analyzed by two-way ANOVA. There was a main effect of treatment, F (1, 36) = 20.20, *p* < 0.0001, but not sex on the innervation/tumor volume ratio. There was no interaction between sex and treatment (Fig. 8C); post-hoc Tukey’s test confirmed the treatment effect.

**Fig. 9:**
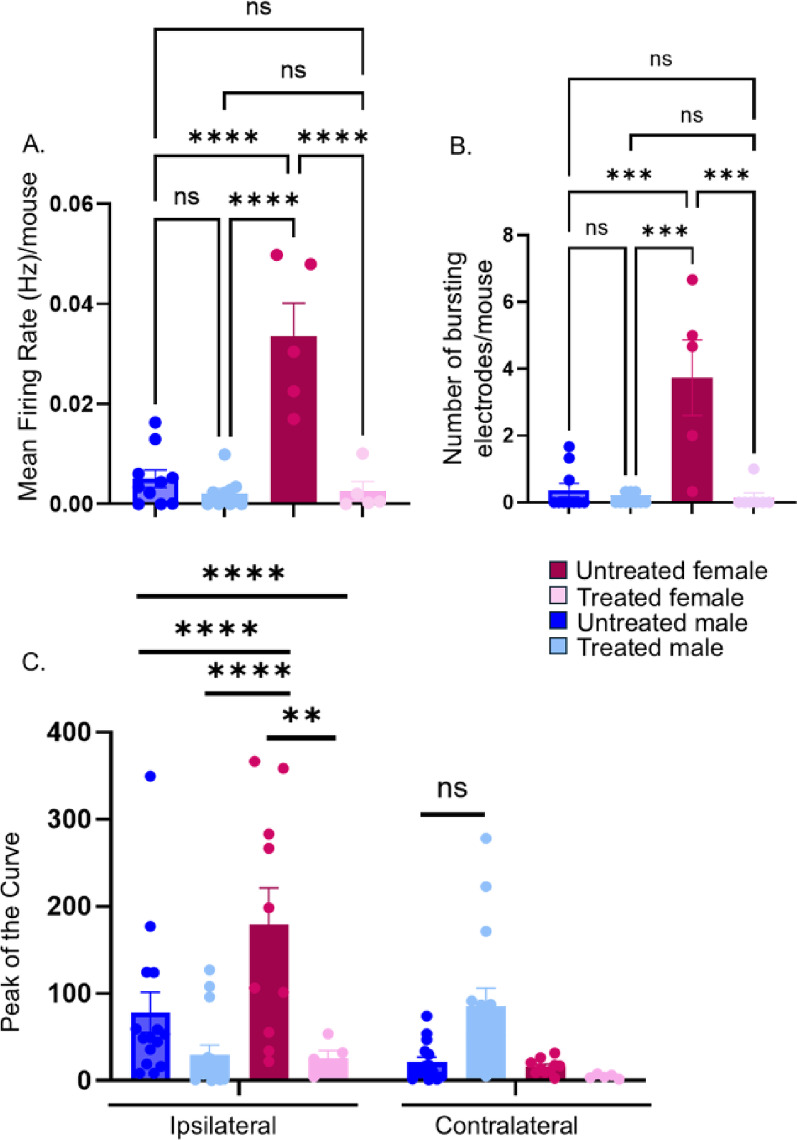
Cisplatin/radiation treatment attenuates activity of intra-tumoral nerves. Tumors from male and female mice (untreated vs. cisplatin/radiation treated) were harvested, sliced and analyzed by microelectrode array (*n* = 5 females/group; *n* = 10 males/group). MEA recordings from 16–27 tumor slices/group were analyzed. Each dot represents the average signal per animal. **A**) Mean firing rate and **B**) number of bursting electrodes were compared between the groups. Statistical analysis by 2-way ANOVA with post-hoc Tukey’s test. For both measures, there is a significant interaction of sex and treatment. **C**) Trigeminal ganglia (ipsilateral and contralateral) from MOC2-7 oral tumor-bearing mice were harvested, dissociated and loaded with calcium indicator, Fluo-4AM. Neurons were stimulated with a TRPV1 agonist (capsaicin, 300nM) and fluorescence quantified and compared between the groups. Statistical analysis by 3-way ANOVA with Tukey’s multiple comparisons test. Calcium imaging studies were repeated in a separate cohort of tumor-bearing mice with similar results (data not shown). *, *p* < 0.05; **, *p* < 0.01; ****, *p* < 0.0001; ns, not significant. Error bars, SEM

Using microelectrode arrays (MEA), we previously demonstrated that tumor-infiltrating nerves remain functional at the tumor bed [17]. The paradoxical increase in innervation density following cisplatin/radiation therapy in the present study, led us to hypothesize that tumor-infiltrating nerves in post-treatment tumors harbor increased electrical activity compared to pre-treatment nerves. To test this, male and female mice were again implanted with oral MOC2-7 tumors and treated with cisplatin/radiation or vehicle as described. On day 28 post-tumor implantation, tumors were extracted, sectioned, and analyzed by MEA. Similar to our published study, tumors harvested from control (no chemoradiotherapy) animals were electrically active. Consistent with their increased nerve density, female tumors had significantly increased electrical activity as compared to male tumors. This was evident in the mean firing rate (Fig. 9A). Two-way ANOVA revealed that there was a main effect of sex, F (1, 26) = 27.66, *p* < 0.0001, as well as of treatment, F (1, 26) = 38.06, *p* < 0.0001. Post-hoc Tukey’s test revealed treated females had lower firing rates than untreated females (*p* < 0.0001; Fig. 9A). Similarly, two-way ANOVA of bursting electrodes revealed main effects of Treatment (F (1, 25) = 19.01, *p* = 0.0002) and Sex (F (1, 25) = 22.29, *p* < 0.0001), with a significant Sex × Treatment interaction (F (1, 25) = 22.29, *p* < 0.0001; Fig. 9B). Contrary to our hypothesis, tumors harvested post-therapy had significantly less electrical activity compared to pre-treatment, though this decrease was only observed in female tumors (Fig. 9A, B). Contrary to our hypothesis, however, tumors harvested post-therapy had significantly less electrical activity as compared to pre-treatment (Fig. 9A, B) though this was only true for female tumors.

While intriguing, MEA measures the electrical activity of all cells captured in the tumor slice, not only nerves. To specifically assess the activity of tumor-infiltrating neurons, we initiated calcium imaging studies. Here, the ipsilateral trigeminal ganglia from tumor-bearing mice were harvested, dissociated, and loaded with a fluorescent calcium indicator. Calcium indicators are widely used in neuroscience to quantify neuronal activity as they fluoresce following an influx of calcium. We have previously utilized calcium indicators to show that tumor-infiltrating nociceptors from the ipsilateral trigeminal ganglia have significantly higher calcium responses to the TRPV1 agonist, capsaicin, as compared to control (non-tumor) trigeminal neurons or those from the contralateral side of tumor-bearing animals [36]. A three-way ANOVA revealed significant main effects of side (*P* = 0.0049) and treatment (*P* = 0.0230), as well as significant side × sex (*P* = 0.0054), side × treatment (*P* = 0.0002), and sex × treatment (*P* = 0.0064) interactions, indicating that the effects of tumor side and treatment on trigeminal neuron calcium signaling were dependent on sex and treatment condition (Fig. 9C). Post-hoc multiple comparisons showed that treatment significantly decreased neuronal activity in males and females. In the absence of chemoradiotherapy, ipsilateral trigeminal neurons responded to capsaicin with a significantly higher calcium influx as compared to those from the contralateral side, which served as controls (Fig. 9C). This is consistent with our prior published findings [36]. However, ipsilateral trigeminal neurons harvested from chemoradiation-treated animals responded to agonist with significantly lower calcium influx as compared to that from contralateral neurons (Fig. 9C). These findings are consistent with the MEA data showing an overall decrease in tumoral electrical activity following chemoradiotherapy (Fig. 9A, B). Interestingly, the contralateral trigeminal neurons from males responded to agonists with higher calcium activity as compared to their counterparts from untreated animals, though this was not statistically significant. In females, the contralateral neurons showed minimal response to agonist (Fig. 9C).

**Fig. 10:**
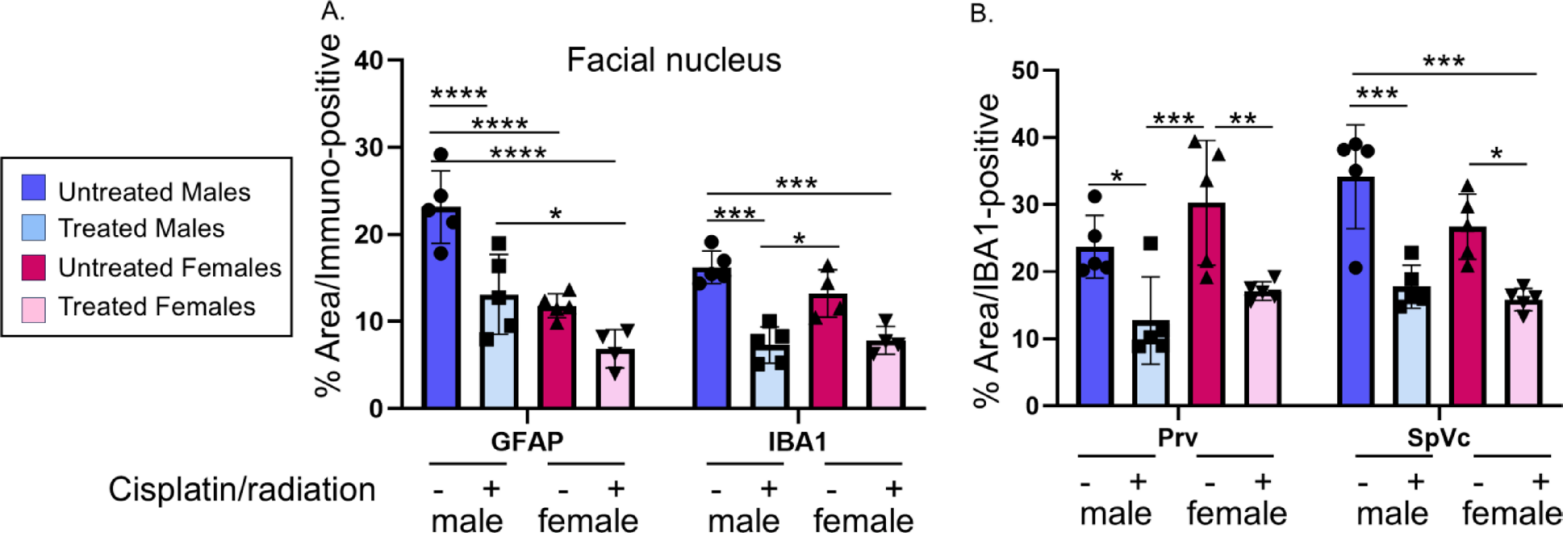
Tumor-induced central glial activation is attenuated by cisplatin/radiation treatment. Brains from male and female mice (control vs. cisplatin/radiation treated) were harvested, sectioned and immunofluorescently stained for markers of astrocytic (GFAP) and microglial (IBA1) activation. At least *N* = 3 sections/mouse/brain region/group were analyzed. *N* = 4–5 mice/group. Statistical analysis by 2-way ANOVA with post hoc Tukey’s test. **A**) Quantification of GFAP (astrocytes) and IBA-1 (microglia) within the facial nucleus. **B**) Quantification of IBA-1 in the principal sensory nucleus (Prv) or the spinal nucleus (SpVc) of the trigeminal. *, *p* < 0.05; **, *p* < 0.01; ***, *p* < 0.005; ****, *p* < 0.0001. Error bars, SD

### Chemoradiation treatment attenuates glial and neuronal activation in the brains of tumor-bearing animals

**Fig. 11:**
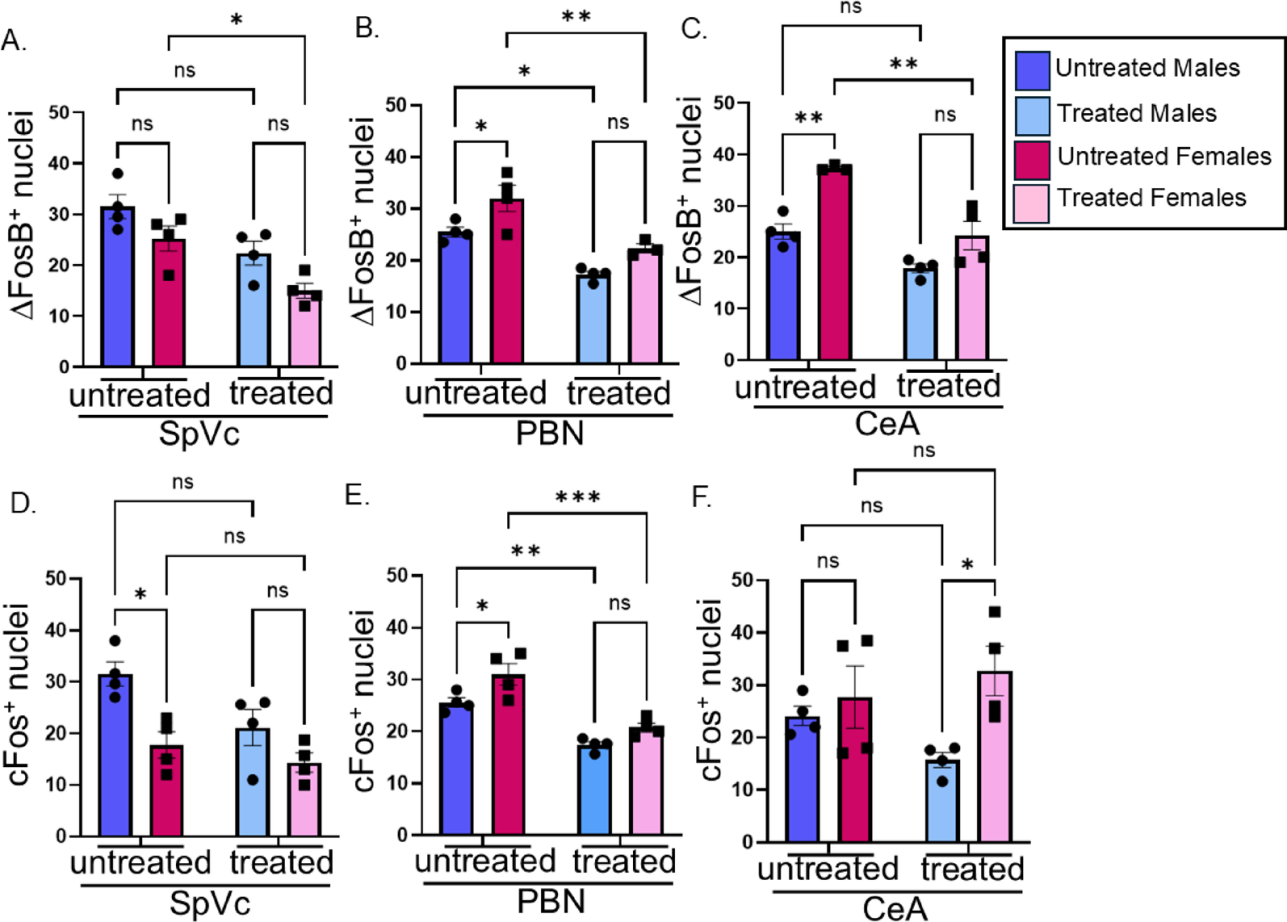
Central neuronal activation in tumor-bearing mice. Brains from male and female mice (control vs. cisplatin/radiation treated) were harvested, sectioned and immunofluorescently stained for cFos and ΔFosB, markers of neuronal activation. At least *N* = 3 sections/mouse/brain region/group were analyzed. ΔFosB (**A-C**) and cFos (**D-F**) quantification in the spinal nucleus of the trigeminal (SpVc), the central amygdala (CeA), and the parabrachial nucleus (PBN). *N* = 4–5 mice/group. Statistical analysis by 2-way ANOVA with post hoc Tukey’s test. *, *p* < 0.05; **, *p* < 0.01; ***, *p* < 0.005; ****, *p* < 0.0001

Oral cavity tumors are known to be painful [68–70], and it is increasingly understood that resident glial cells in the brain, astrocytes (the most abundant glial cells in the brain) and microglia (the brain’s immune cells), actively contribute to central changes associated with pain [71]. In fact, we have shown that oral MOC2-7 tumors cause pain in mice which negatively impacts nesting behavior and that treating this pain with carprofen (a non-steroidal anti-inflammatory) completely restores nesting [36]. Given the tumor-brain circuit and the ability of chemoradiotherapy to ameliorate nesting behavior (Fig. 7A), we assessed the brains of tumor-bearing male and female mice for central glial activation, focusing specifically on astrocytes and microglia. Astrocyte activation was assessed by immunofluorescent staining for GFAP in the facial nucleus as well as the primary and sensory nuclei of the trigeminal. Following chemoradiotherapy, GFAP was significantly higher only in the facial nucleus in treated males compared to treated females. Specifically, 2-way ANOVA revealed a main effect of sex (F [3, 29] = 31.75, *p* < 0.0001) (Fig. 10A). The most interesting finding was that in all brain regions assessed [facial nucleus (7 N), primary nucleus of the trigeminal (SpVc) and sensory nucleus of the trigeminal (PrV)], chemoradiotherapy significantly attenuated activation of both astrocytes (GFAP) and microglia (for IBA-1) in males and females (2-way ANOVA; main effect of treatment, F [3, 29] = 31.75, *p* < 0.0001) (Fig. 10A, B). This decrease in glial activation, together with the decreased neuronal activity of tumor-infiltrating TGM neurons (Fig. 9C), suggests that chemoradiotherapy attenuates activity of the tumor-brain circuit despite the local increase in tumor innervation in the periphery (Fig. 8A-C). To further assess this, we immunofluorescently stained brains for cFos and ΔFosB, two neuronal transcription factors that serve as surrogates for neuronal activity [72]. Three brain regions within the tumor-brain circuit were assessed: the SpVc, the parabrachial nucleus (PBN) and the central amygdala (CeA). Two-way ANOVA revealed a main interactions of sex and treatment in all brain areas for both cFos and ΔFosB expression (all *p* < 0.05). However, sex x treatment interactions were not significant for any brain region or marker (all *p* > 0.05). Specifically, untreated females had significantly higher ΔFosB expression in the PBN and CeA (*p* < 0.01). Chemoradiotherapy significantly attenuated ΔFosB expression in the PBN of males and in all three brain regions of females (SpVc, PBN, CeA; all *p* < 0.05; Fig. 11A-C). Untreated males had significantly higher cFos expression in the SpVc (*p* < 0.05), while untreated females had higher cFos in the PBN (*p* < 0.05; Fig. 11D, E). Cisplatin/radiation treatment significantly decreased cFos expression in the PBN of both sexes (*p* < 0.001) but had no effect in the SpVc or CeA (Fig. 11D-F). Overall, chemoradiotherapy attenuated activity of the tumor-brain circuit more extensively in females (evidenced in SpVc, PBN, and CeA) compared to males (where attenuation was evident only in the PBN). Given that untreated female tumors are significantly more innervated than males (Fig. 2A-C), these findings suggest that densely innervated tumors may have an increased influence on neuronal activity in projection areas. Moreover, these data show that despite increasing tumor innervation at the tumor bed, cisplatin/radiation treatment attenuates activity of the tumor-brain circuit as well as of tumor-infiltrating neurons (Fig. 9). Taken together, these data show an overall trend for decreased glial and neuronal activation in the tumor-brain projection areas following treatment with cisplatin/radiation. This correlates with the decreased activity (as measured by MEA and Ca^+ 2^ signaling) of peripheral TGM neurons on the ipsilateral side (Fig. 9). The improvement of nesting behavior (Fig. 7) following treatment suggests that this attenuation in neuronal activity contributes to the restoration of baseline behavior.

**Fig. 12:**
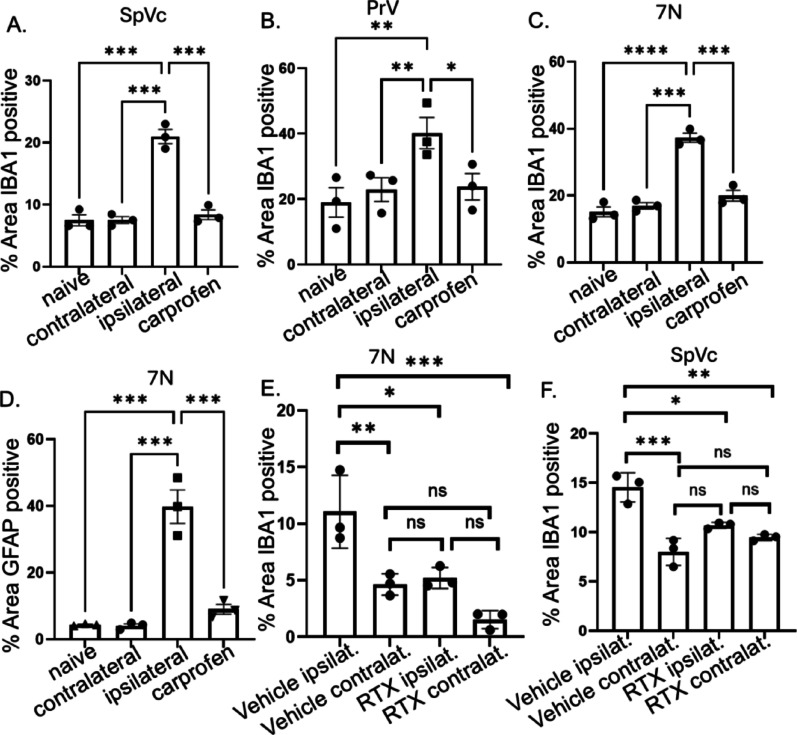
Carprofen and TRPV1 neuronal ablation attenuate glial activation in the brains of tumor-bearing animals. Mouse brains from a previous study [31] were analyzed for glial activation. In that study, male C57BL/6 mice were implanted with MOC2-7 cells. Mice were treated with vehicle or carprofen (10 mg/kg) provided in the drinking water. Twenty-five days post-tumor implantation, animals were euthanized, brains harvested, and immunofluorescently stained. IBA-1 microglial staining in the spinal nucleus of the trigeminal (A), sensory nucleus of the trigeminal (B), and facial nucleus (C). GFAP astrocyte staining for the facial nucleus (D). *N* = 9 or 10 mice/group; *n* = 3 brains/condition with *n* = 3–4 slices/brain stained. Brains from another study of male mice with oral MOC2-7 tumors were analyzed for IBA-1. Prior to tumor implantation, mice were treated with RTX (to ablate TRPV1 neurons) or vehicle (intact neurons). IBA-1 staining in the facial nucleus (E) and spinal nucleus of the trigeminal (F) was quantified. *n* = 4–6 mice/group with *n* = 3–4 slices/mouse. Each dot represents the average of 2–3 slices per mouse. Statistical analysis by 2-way ANOVA with post-hoc comparisons. * *p* < 0.05; ** *p* < 0.01; *** *p* < 0.001; ns, not significant. Error bars, SEM

### Carprofen treatment attenuates glial activation in the brains of tumor-bearing animals

Given that glial activation has already been associated with peripheral pain [71], we wondered whether tumor-associated pain signals from the periphery were responsible for the glial activation in the brains of tumor-bearing animals. To address this question, we assessed the brains from a previous study in which MOC2-7 oral tumor-bearing male mice had been treated with or without carprofen, a non-steroidal anti-inflammatory commonly used to treat pain. In that study, carprofen treatment fully restored nesting behavior [36]. Additional control brains from naïve (non-tumor) animals were also analyzed. Activation of microglia in naïve animals was low and not significantly different from that of the contralateral side of tumor-bearing animals. This was true in all the brain regions assessed: SpVc, PrV and 7 N. Microglial activation was significantly increased on the ipsilateral, but not the contralateral side, in the brains of tumor-bearing animals, and this activation was attenuated to naïve levels with carprofen treatment (two-way ANOVA; main effects of Side and Treatment in SpVc, PrV, and 7 N: all *p* < 0.05), whereas contralateral microglial activation did not differ from naïve levels (Fig. 12A-C). When astrocyte activation was assessed, only the facial nucleus showed significantly increased staining on the ipsilateral side which was attenuated by carprofen treatment (two-way ANOVA; main effects of Side and Treatment: *p* < 0.001; Fig. 12D). Taken together, these data indicate that the presence of a peripheral oral tumor increases ipsilateral activation of glial cells centrally. Since pain treatment (carprofen) attenuated glial activation to baseline levels (e.g., naïve and contralateral), the data suggest that glial activation correlates with oral pain induced by the tumor. Consistent with this, and similar to carprofen’s effect on nesting behavior in tumor-bearing mice [36], we found that treatment with cisplatin and radiation also attenuates neuronal activation in the brain (Fig. 11), partially restores nesting (Fig. 7A).

 Inflammatory cytokines are known to activate central glia [67, 73]. In fact, soluble inflammatory factors released into circulation from the tumor microenvironment may contribute to glial activation in the CNS [74]. However, given the tumor-brain circuit, we asked whether, in addition to inflammatory cytokines, the direct neural link from tumor to brain may also impact activation of central glia in the context of an oral tumor. To address this, peripheral TRPV1-expressing sensory neurons (previously defined to innervate oral tumors) [13, 75] were chemically ablated with resiniferatoxin (RTX), a potent analog of the TRPV1 agonist, capsaicin [76, 77]. Since these are the same neurons that infiltrate oral tumors, implantation of tumors into RTX-ablated animals compromises the tumor-brain circuit. Glial activation was assessed in the brains of tumor-bearing RTX-ablated and control (intact) animals. Microglial and astrocyte activation was significantly attenuated in the brains of ablated, tumor-bearing animals (2-way ANOVA; main effects of neuronal ablation in 7 N and SpVc: all *p* < 0.05; Figure 12E, F). These data link TRPV1 tumor-infiltrating nerves in the periphery with tumor-associated glial activation centrally and suggest signals from oral tumors relayed centrally, via the tumor-brain circuit, contribute to tumor-associated glial activation.

**Fig. 13:**
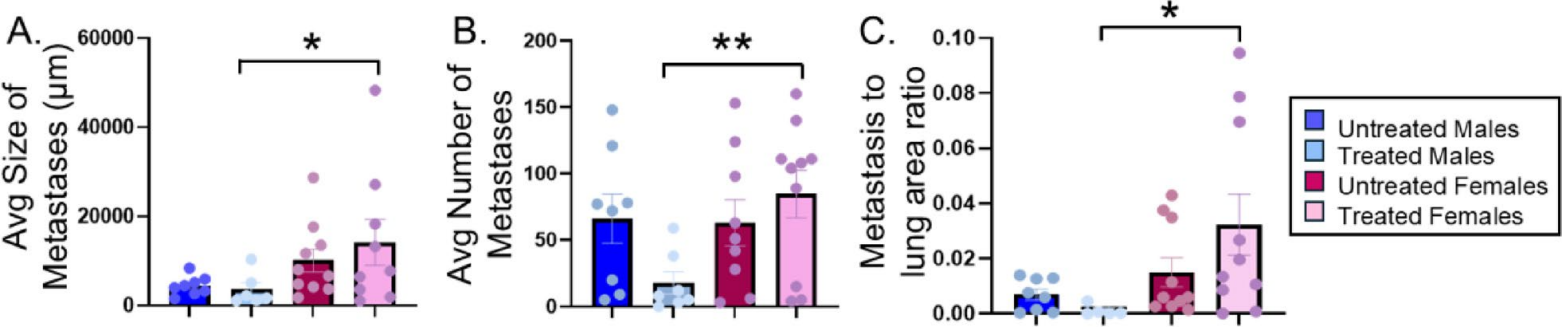
Influence of sex on metastasis. The lungs from MOC2-7 oral tumor-bearing male and female mice were immunohistochemically stained for cytokeratin and metastasis quantified. The average size (**A**), number (**B**), and metastasis-to-lung area (**C**) between males and females were compared. Statistical analysis was performed by two-way ANOVA with post-hoc Fisher’s LSD test. *N* = 7–10 mice/group. *p < 0.05, **p < 0.01. Asterisks indicate post-hoc comparisons between treated males and treated females. See text for main effects

### Sex differences in metastasis

The sex differences in tumor innervation that we observed at the primary site, prompted us to ask whether they impact metastasis. Given that oral MOC2-7 tumors metastasize to the lung [78], these organs were harvested upon euthanasia and immunohistochemically stained for cytokeratin to identify metastases. Analysis of the size, number and metastasis-to-lung area ratio shows that while there is a significant effect of sex on size of metastatic lesions (*p* = 0.0219, F (1, 29) = 5.870] as well as the lesion-to-lung area ratio [*p* = 0.0379, F (1, 30) = 4.716], there was no effect of sex on the number of lesions. However, a significant Sex × Treatment interaction was observed for lesion number (F (1, 30) = 4.23, *p* = 0.048; Fig. 13A-C). Post-hoc analysis revealed that treated females had significantly more metastatic lesions (*p* = 0.010) and larger lesions (*p* = 0.042) compared to treated males, resulting in a higher metastasis-to-lung area ratio (*p* = 0.033). These data suggest that female mice were resistant to cisplatin/radiation therapy whereas males responded with a significantly lower metastatic burden. While strictly correlative, our data suggest that the treatment-associated increased innervation in female tumors (Fig. 8) may contribute to the higher metastatic burden evident in treated females.

## Discussion

We show that oral MOC2-7 tumors grow significantly slower in females yet are more densely innervated than their male counterparts. We also found this sex difference in mEERL95 (HPV+ HNSCC model) and a similar trend with YUMMER1.7 (melanoma model) tumors suggesting this may be a general phenomenon. While the decreased tumor growth in females with YUMMER1.7 melanoma may be caused by a sex-induced immune response rather than a bona fide sex difference in tumor growth, the increased innervation cannot be explained by this mechanism. Many publications (our own included) indicate that increased nerve density correlates with faster, not slower, tumor growth [17, 79–83]. However, studies specifically assessing tumor innervation between males and females are quite limited; this study addresses this knowledge gap.

The finding that female tumors grow slower and yet are more densely innervated than tumors in males suggests that that tumor microenvironments generated in females and males are different. The importance of sex differences in immune cell functions are increasingly recognized as critical factors in health and disease. In addition to differing by sex, immune cell functions fluctuate with age, developmental stages (e.g., puberty) and reproductive status (e.g., pregnancy, menopause). These sex differences are reflected in sex disparities observed in different diseases, including cancer [84–89]. Consistent with this, analysis of cancer mortality (for 20 different cancers) showed that females have a 27% lower risk of dying from cancer as compared to males [89]. Gene Set Enrichment Analysis of publicly available datasets of different cancer types showed that immune processes in females with head and neck cancer were higher than in males; these processes included immune system development, immune response, activation of immune response and response to stimuli [89]. These and other findings [84, 90] suggest that smaller MOC2-7 tumors in female mice reflect their more robust anti-tumor immune response. Though studies focused on sex differences are few, it is important to note that two publications found no differences in tumor growth using orthotopic (tongue) implantation of MOC1 and MOC2 head and neck cancer cell lines [91, 92]. While these publications implanted cells in the tongue (as opposed to the oral cavity, as in this current study), a key methodological difference may explain the discrepancy between our findings. The two studies which showed no sex difference in tumor growth included Matrigel with tumor cell implantation, we did not. This basement membrane extract contains a mixture of several extracellular matrix proteins and growth factors which may generate a rich environment conducive for tumor growth. Moreover, inclusion of Matrigel provides an immune barrier [93] and/or enables more reliable tumor-take [94, 95], either of which could mask potential sex differences in tumor growth.

Increased innervation in smaller female tumors suggests that nerve infiltration of malignancies is an early event. We speculate that in males, where tumor growth is sustained, the proliferative rate of tumor cells outpaces the recruitment of nerves resulting in a lower density of axons in larger male tumors. It is also possible that male tumors actively exclude or promote retraction of nerves (these possibilities are not mutually exclusive). Studies have, in fact, demonstrated that the infiltration of tumors by axons is an early event in some cancers, including head and neck [36, 96]. Despite this sex difference in tumor growth and innervation, there was no influence of sex on nesting, running or burrowing performance suggesting that the sex difference is local (restricted to the tumor bed) and does not extend centrally. Consistent with this, tracing studies indicate that the anatomical tumor-brain circuit is qualitatively similar in males and females, though females exhibited greater neuronal activation in brain projection areas (Fig. 11). Importantly, the behaviors assayed here do not assess pain. The increased tumor innervation in females likely results in increased pain. Consistent with this, two tongue cancer models (MOC1, MOC2) showed increased nociceptive behavior in tumor-bearing females [91]. While tumor innervation was not assessed, our data suggest that the increased pain exhibited by females was likely due to increased innervation of their malignancies.

We show that chemoradiotherapy increased innervation of female tumors but that these infiltrating nerves were hypoexcitable. Moreover, following cisplatin/radiation treatment, females had increased metastases in their lungs compared to treated males. Consistent with this latter finding, analyses of the Cancer Genome Atlas [97] and SEER database [98] showed that female patients experience significantly more metastatic lung disease compared to males. While our findings are purely correlative, they provide a foundation to test mechanistic pathways driving these sex differences.

It is important to note that chemoradiotherapies are well documented to injure nerves. In fact, chemotherapy-induced peripheral neuropathy is commonly experienced by cancer patients [99, 100]. Cisplatin, a standard-of-care treatment for head and neck cancers, induces demyelination of Schwann cells, thus increasing the vulnerability of nerves and axons to degeneration [101]. Radiation can induce oxidative stress, apoptosis, and neuroinflammation that also contribute to neuropathy [102]. Nerve damage induced by local radiation likely activates a program of nerve regeneration, which could enhance innervation post-treatment. Consistent with this, low-dose X-ray irradiation after peripheral nerve injury in rats increased VEGFA and GAP-43 protein levels, which are involved in axon regeneration and myelination [103]. Similarly, gamma knife irradiation, a precise, minimally invasive form of radiotherapy used to treat tumors, was found to elevate β-III tubulin protein expression, suggesting it may mediate nerve regeneration and injury repair [104]. We and others have shown that tumor-infiltrating neurons express the *Atf3* transcription factor [91, 105–107]. This transcription factor orchestrates the expression of Regeneration Associated Genes (RAGs) that induce robust extension of neurites necessary for repair. Part of this repair program includes early suppression of synaptic transmission and neural network activity to allow time for restoration of damaged axons. Within hours to days post-injury, however, peripheral neurons become hyperexcitable [108], a state that can become sustained over time [109]. Taken together, our current findings suggest that cisplatin/radiation treatment injures tumor-infiltrating nociceptors, thereby initiating the *Atf3*-RAGs repair program and that this effect is more robust in females. Within the tumor microenvironment, this injury may initially suppress neuronal activity while simultaneously potentiating innervation. Interestingly, estrogen can promote nerve regeneration [110, 111] which may explain, in part, the sex difference we observed. Over time and as nerve density increases, neurons may shift to a hyperexcitable state. This potentiated release of neurotransmitters and neuropeptides likely contributes to disease progression, including metastasis. While this hypothesis requires testing, our correlative findings serve as a starting point to mechanistically define sex differences in tumor innervation and its consequences.

While chemoradiotherapy slows tumor growth and benefits patients in the short term, our study suggests a paradoxical consequence: potentiation of innervation in residual and/or recurrent disease. This study also suggests that adding nerve blockers or blockers of nerve growth to this standard-of-care treatment may circumvent its influence on nerves. Our findings also suggest that female patients are likely to be exquisitely responsive to nerve blocking treatments. However, to assess the potential clinical relevance of our findings, studies analyzing sex differences in tumor innervation are paramount. Such efforts will require large patient cohorts, especially in head and neck cancer where females make up a minority of the patient population [27, 112]. The ability to slow tumor growth in conjunction with blocking tumor innervation is likely to further attenuate, or reverse, cancer-associated behavioral declines. While such a therapeutic strategy is not yet clinically available, this study, along with others, continues to indicate that targeting nerves in cancer therapies will be the next generation of treatments added to the arsenal for treating and curing cancers.

Though informative, this study is not without limitations, perhaps the biggest one being that the data are a collection of correlations. We do not provide any definitive mechanistic data supporting our speculations but the data do provide provocative sex differences that can now be mechanistically defined. For example, if sex-dependent immune responses at the tumor bed explain the smaller tumor sizes in females, this sex difference should be eliminated when tumors are implanted into immune incompetent animals. Moreover, if initiation of the neural repair *Atf3*-driven transcriptional program predominantly leads to increased tumor innervation post-treatment, this effect should be eliminated when tumors are implanted into *Atf3* knock-out animals. In addition, if increased lung metastasis in tumor-bearing female mice post-treatment is a consequence of neuronal hyperactivity, silencing these signals (e.g., Exparel^®^, a voltage-gated Na+ channel blocker) could attenuate distant metastasis. While more research is necessary, this study highlights the influence of sex on tumor innervation, tumor growth, and treatment response.

## Supplementary Information

Supplementary Material 1.

## Data Availability

The datasets used and/or analyzed during the current study are available from the corresponding author on reasonable request.
