# Supplementary material for: Sex influences on tumor innervation
Source: Biol Sex Differ. 2026 Apr 10;17:111. doi: 10.1186/s13293-026-00887-9 (PMC13214194; doi:10.1186/s13293-026-00887-9)
Supplement: Supplementary file 1 — Supplementary Material 1. [file 13293_2026_887_MOESM1_ESM.docx]

**Title: Sex Influences on Tumor Innervation**

**Authors:** Sarah M. Barclay^1,2^, Jeffrey Barr^1^, Mangalam Bajpai^1,2^, Oduduabasi Isaiah^1^, Hailey Bullard^3^, Ethan Neufeld^4^, Craig Welbon^1^, Payal Ghosh^1^, Destiny S. Brockhaus^1,2^, William C. Spanos^1,5^, Paola D. Vermeer^1,2,5^

Supplemental Figures


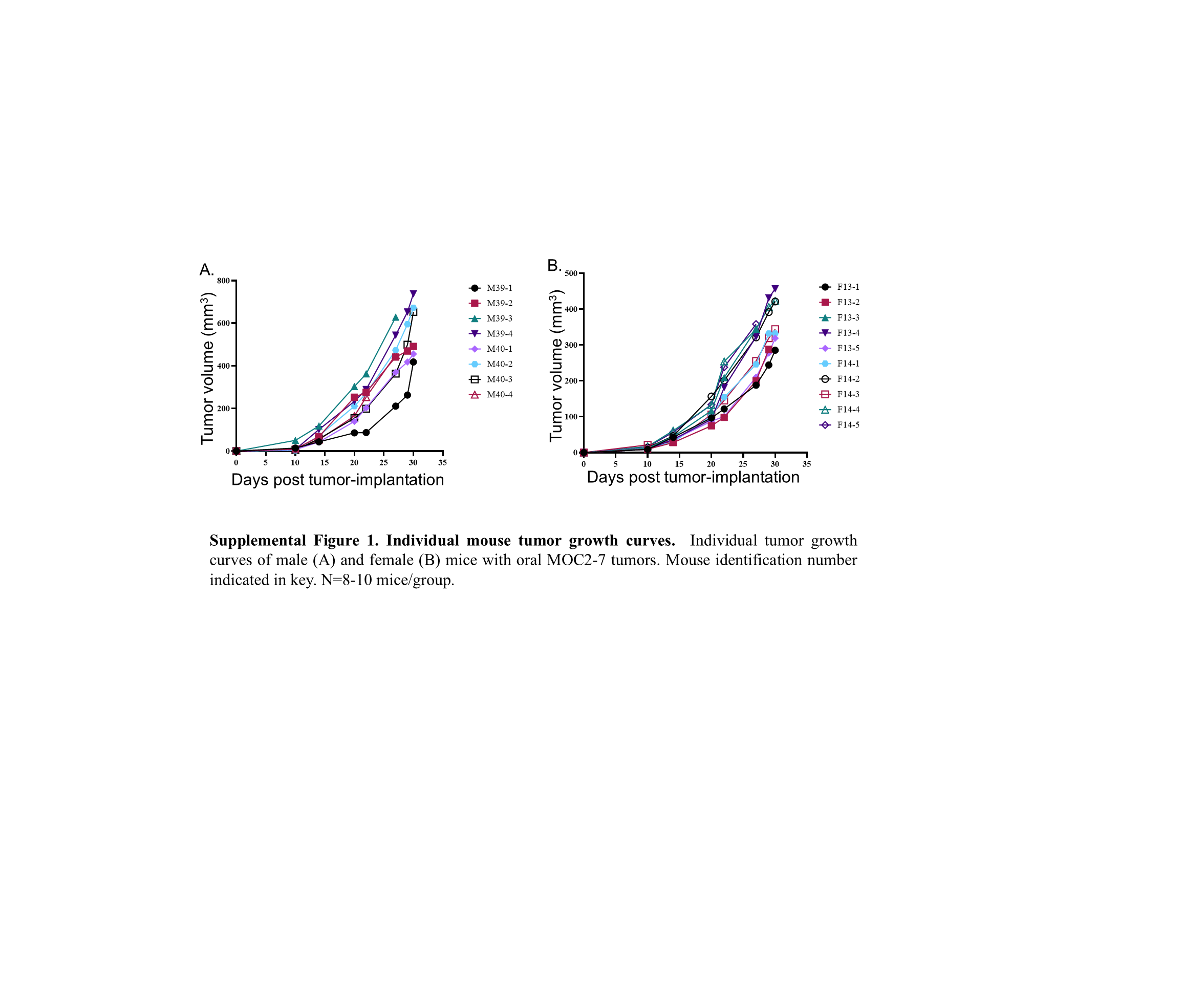


**Supplemental Figure 1. Individual mouse tumor growth curves.**  Individual tumor growth curves of male (A) and female (B) mice with oral MOC2-7 tumors. Mouse identification number indicated in key. N=8-10 mice/group.


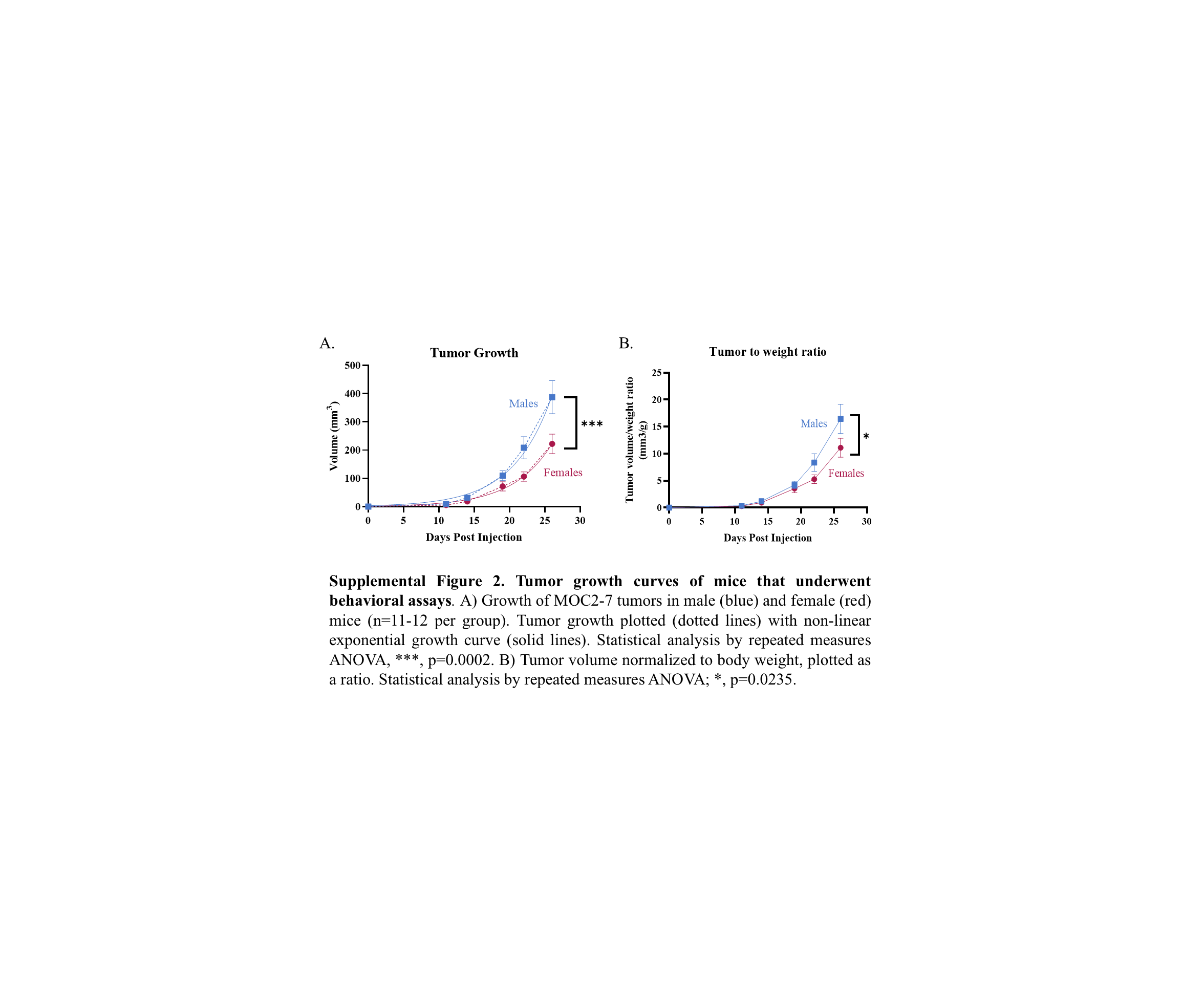


**Supplemental Figure 2. Tumor growth curves of mice that underwent behavioral assays***.* A) Growth of MOC2-7 tumors in male (blue) and female (red) mice (n=11-12 per group). Tumor growth plotted (dotted lines) with non-linear exponential growth curve (solid lines). Statistical analysis by repeated measures ANOVA, ***, p=0.0002. B) Tumor volume normalized to body weight, plotted as a ratio. Statistical analysis by repeated measures ANOVA; *, p=0.0235.


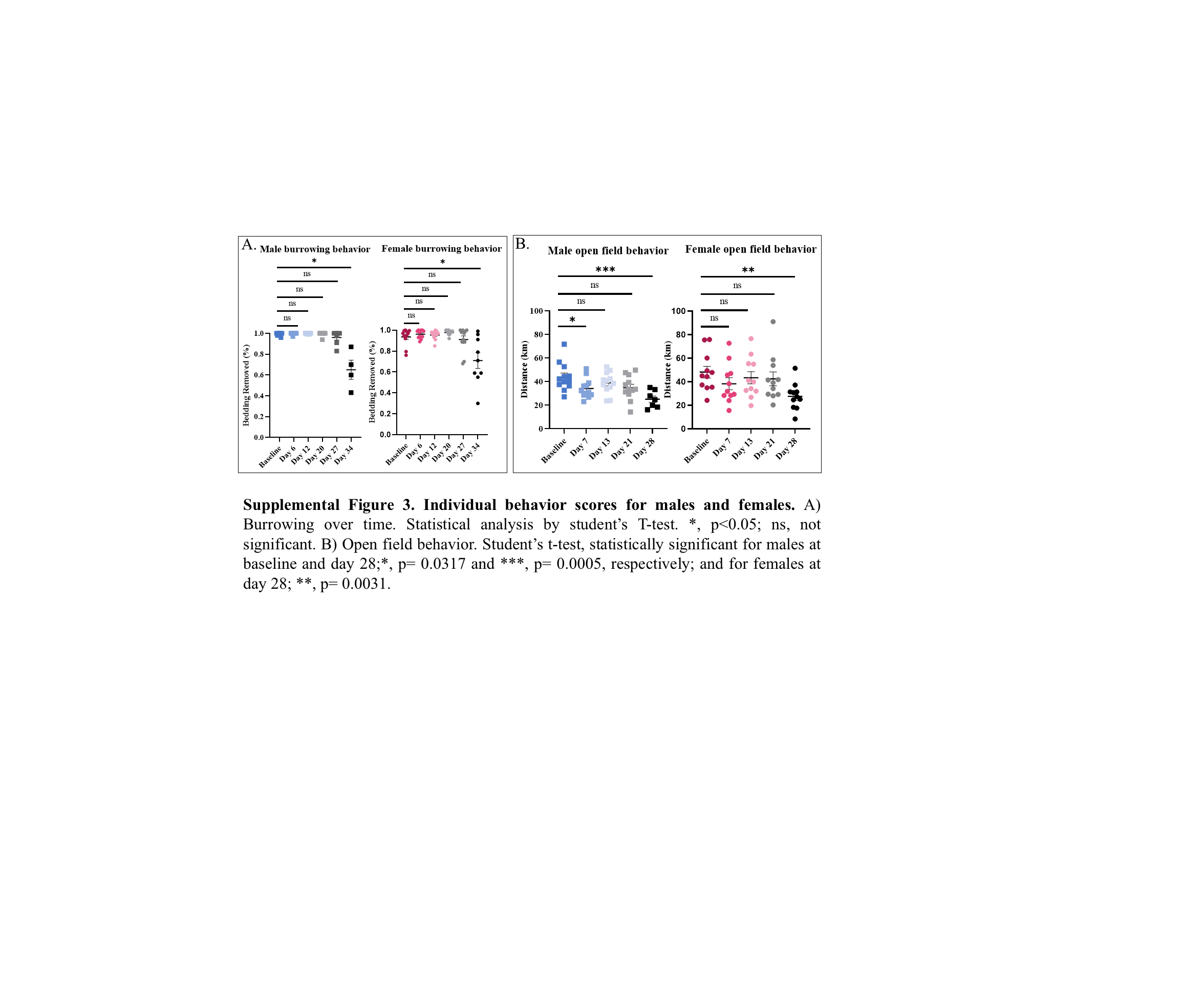


**Supplemental Figure 3. Individual behavior scores for males and females.** A) Burrowing over time. Statistical analysis by Student’s t-test for each sex is shown separately. *, p<0.05; ns, not significant. B) Open field behavior. Student’s t-test for each sex is shown separately. *, p<0.05; **, p< 0.01; ***, p< 0.005.


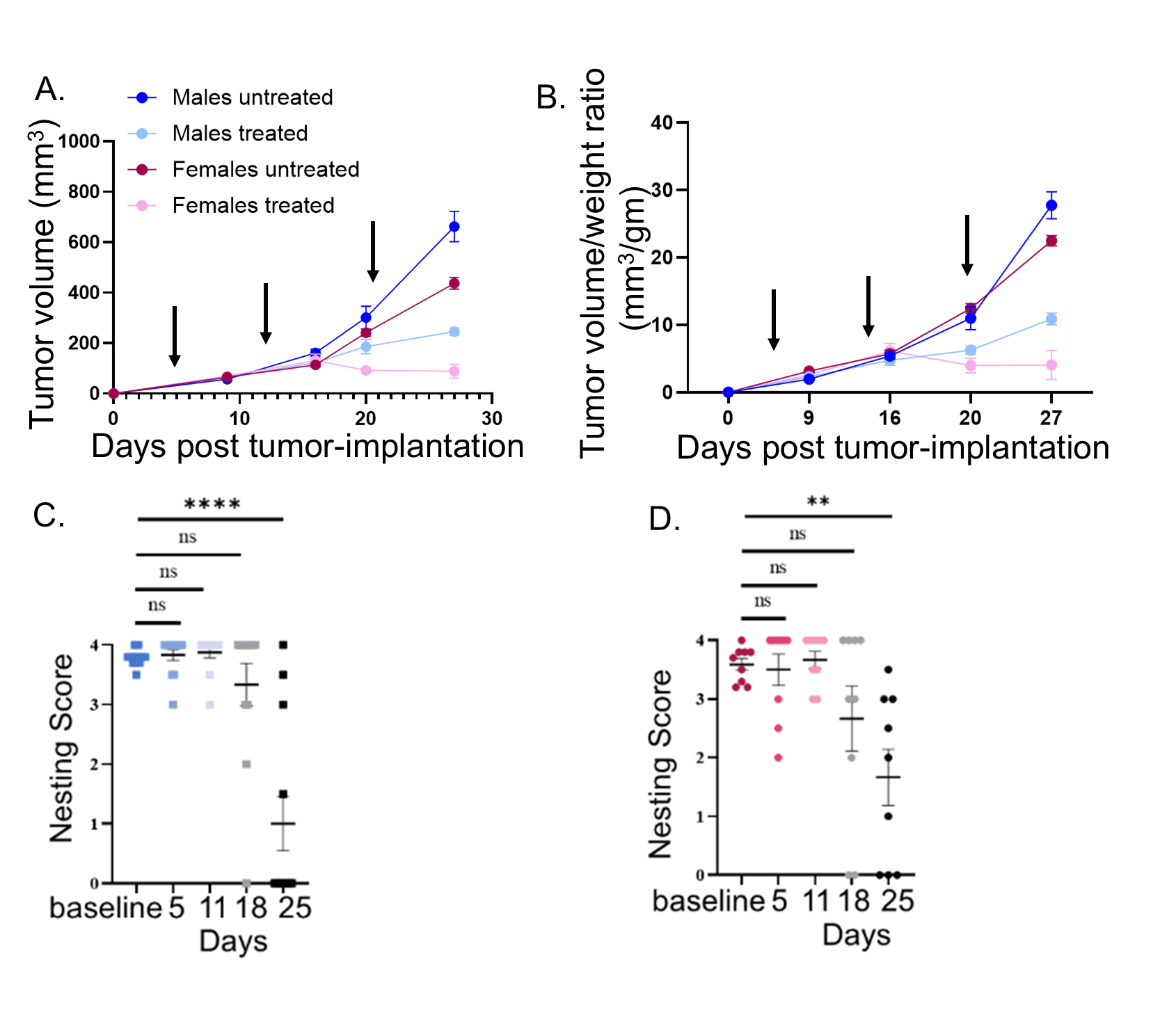


**Supplemental Figure 4. Sex influences tumor growth.** Tumor volume of male (A) and female (B) mice, (n=10 mice/group) treated with or without cisplatin and radiation on days 8, 15 and 22 post tumor implantation. Statistical analysis by three-way ANOVA. There was a significant effect of time, F(2.158, 53.94) = 154.6, p<0.0001; there was a significant effect of sex, F(1, 25 0= 70.61, p<0.0001, however there was no significant effect of treatment. There was a significant interaction between time and treatment, F(2.158,53.94)=8.093, p=0.0006 and also between time and sex, F(2.158, 53.94)= 57.31’ p<0.0001.
